# Time optimal entrainment control for circadian rhythm

**DOI:** 10.1371/journal.pone.0225988

**Published:** 2019-12-18

**Authors:** A. Agung Julius, Jiawei Yin, John T. Wen

**Affiliations:** 1 Dept. Electrical, Computer, and Systems Engineering, Rensselaer Polytechnic Institute, Troy, NY, United States of America; 2 Lighting Enabled Systems and Applications (LESA) Engineering Research Center, Rensselaer Polytechnic Institute, Troy, NY, United States of America; 3 Dept. Industrial and Systems Engineering, Rensselaer Polytechnic Institute, Troy, NY, United States of America; Oxford Brookes University, UNITED KINGDOM

## Abstract

The circadian rhythm functions as a master clock that regulates many physiological processes in humans including sleep, metabolism, hormone secretion, and neurobehavioral processes. Disruption of the circadian rhythm is known to have negative impacts on health. Light is the strongest circadian stimulus that can be used to regulate the circadian phase. In this paper, we consider the mathematical problem of time-optimal circadian (re)entrainment, i.e., computing the lighting schedule to drive a misaligned circadian phase to a reference circadian phase as quickly as possible. We represent the dynamics of the circadian rhythm using the Jewett-Forger-Kronauer (JFK) model, which is a third-order nonlinear differential equation. The time-optimal circadian entrainment problem has been previously solved in settings that involve either a reduced-order JFK model or open-loop optimal solutions. In this paper, we present (1) a general solution for the time-optimal control problem of fastest entrainment that can be applied to the full order JFK model (2) an evaluation of the impacts of model reduction on the solutions of the time-optimal control problem, and (3) optimal feedback control laws for fastest entrainment for the full order Kronauer model and evaluate their robustness under some modeling errors.

## 1 Introduction

The circadian rhythm is a mechanism with which living beings can synchronize their biological processes with the light and dark pattern of the terrestrial day [1]. Effectively, the circadian rhythm functions as a master clock that regulates these processes [2]. In humans, the circadian rhythm is heavily linked to various physiological processes, including sleep, metabolism, hormone secretion, and neurobehavioral processes. Disruption of the circadian rhythm is known to have negative impacts on health, ranging from fatigue in travelers with jet-lag to an increased risk of cancer in rotating shift workers [3].

Light is the strongest circadian stimulus. In the literature, there are mathematical models that capture the dynamics of the circadian rhythm and how light affects it. The most detailed models are based on the biochemical and gene regulation processes behind the circadian rhythms, such as those in [4–6]. Empirical models, such as variants of the well-known Jewett-Forger-Kronauer (JFK) model [7, 8], are simpler and capture the essential behavior of the human core body temperature oscillation and the effect of light on the phase and amplitude of this oscillation. As demonstrated in [9], the JFK model may be considered as the asymptotic case of the biochemical models in an averaged sense.

One aspect of circadian rhythm regulation that has received a lot of attention is the (re)entrainment problem, i.e., the problem of driving a misaligned circadian phase to a reference circadian phase. Such problems occur, for example, in travelers with jet-lag or workers with rotating shifts. This problem is typically expressed as an optimal control problem of a system with nonlinear dynamics. The control inputs into the system are typically light [10] and chemicals that target circadian genes [11]. Some researchers have proposed to use model predictive control to deal with the nonlinear dynamics of the circadian rhythm without any guarantee of optimality of the solutions [12–15]. In contrast, others consider the time-optimal control problem related to circadian entrainment [16]. Our prior work (c.f. [17–21]) that used the Pontryagin Maximum Principle approach falls under this category. A related work reported in [22] also posed the time-optimal control problem and solved it by assuming that the optimal light schedule would alternate between using maximally bright circadian light and darkness. In the optimal control literature, such strategies are called *bang-off* strategies. Subsequently, the time-optimal scheduled is computed by optimizing the switching times of the light (i.e., between light on and off).

In this paper, we solve the time-optimal control problem of the fastest entrainment on the JFK model. The JFK model for human circadian rhythm is a third-order nonlinear differential equation, which is detailed in Sec. 2. In our prior work [18, 19], we study the optimal entrainment of a reduced model with second-order dynamics, which is obtained by ignoring a (fast) part of the dynamics, which is called the Process-L. In some later work [20, 21], we study the optimal entrainment of a further reduced model, which is obtained by ignoring the amplitude of the circadian oscillation and focusing on the phase dynamics of the oscillation. These reductions were necessary to solve the time-optimal control problem; otherwise, the solution procedures that we used were not numerically stable. The contributions of this paper can be stated as follows.

- We formulate a general solution for the time-optimal control problem of fastest entrainment that can be applied to the full order JFK model.
- We evaluate the impacts of the ignored dynamics in the reduced-order models on the solutions of the time-optimal control problem.
- We formulate optimal feedback control laws for fastest entrainment for the full order JFK model and evaluate their robustness under modeling error.

The main tool that we use to achieve these results are (i) the lower order models that allow us to compute the solutions of similar (but simpler) problems as initial approximations of the optimal solution, and (ii) the calculus of variations that allows us to formulate a functional gradient descent algorithm to minimize the objective (i.e., entrainment time) from the initial approximations above. Note that the algorithms which are proposed to calculate the (locally or globally) optimal light input for the minimum-time entrainment only depend on the known initial states (open-loop form) instead of the current circadian states during the whole entrainment time (feedback form). If the given values of the initial states are not accurate or disturbances in the light inputs or circadian states occur during the entrainment processes, the minimum-time optimal light inputs which are given as a function of time might turn out to be invalid for entrainment. The feedback implementation of the optimal light input becomes necessary for robust entrainment. To implement the feedback entrainment, we collect data from the computed open-loop optimal trajectories to learn an optimal feedback control strategy. In contrast, existing results to this problem, e.g., those reported in [22], only report on the procedure to obtain the open-loop optimal trajectories. We also demonstrate the robustness of the optimal feedback control strategy by applying it on problem setups that are not used in the training data set.

The rest of the paper is organized as follows. Section 2 covers the full order model and the definition of the time-optimal control problem. In Section 3, we define the reduced-order models and the corresponding time-optimal control problems. In Section 4, we present the algorithms that we use to solve the time-optimal control problems above. In Section 5, we evaluate the impacts of the ignored dynamics in the reduced-order models on the solutions of the time-optimal control problem. In Section 6, we present the optimal feedback control laws for the time-optimal control problem. Finally, Section 7 concludes the paper with some discussion on the main results.

## 2 Problem formulation

The circadian rhythm entrainment problem in this paper is studied based on the JFK model [7]. This model is proposed on the dynamics and light-induced variations in the core body temperature (CBT), comprised with two processes: (1) the Process-L simulates the transduction from the light energy received by the retina to the neuron stimulus transmitted by the retina. It is also closely linked to the light adaption process of our eyes; (2) the Process-P simulates when the SCN receives the stimulus from the retina, how does the CBT change with this light-induced stimulus. The dynamics of the CBT oscillator is formulated and normalized on the Van Der Pol limit cycle. The dynamics of this circadian rhythm model is expressed as the following ordinary differential equations:

$$\frac{dx}{dt}=\frac{\pi }{12}[x_{c}+\mu (\frac{1}{3}x+\frac{4}{3}x^{3}-\frac{256}{105}x^{7})+B],$$ (1)

$$\frac{dx_{c}}{dt}=\frac{\pi }{12}[qx_{c}B-{\left(\frac{24}{0.99729\tau_{x}}\right)}^{2}x-kxB],$$ (2)

$$\frac{dn}{dt}=60(\alpha (I)(1-n)-\beta n),$$ (3)

$$\alpha (I)=\alpha_{0}\cdot {\left(\frac{I}{I_{0}}\right)}^{p},$$ (4)

u=Gα(I)(1-n), (5)

$$B=\left(1-0.4x\right)\left(1-k_{c}x_{c}\right)u.$$ (6)

Here *x*(*t*) and *x*_*c*_(*t*) are the states of the CBT oscillator, and *n*(*t*) is the state of the process that represents retinal photoreceptor saturation (termed *Process-L*). The states *x*(*t*) and *n*(*t*) are normalized and do not have physical units. The unit of *x*_*c*_(*t*) is h^−1^. Light input that enters the receptor is represented by its intensity *I*(*t*), which drives the Process-L. The signal *u*(*t*) is the input to the circadian oscillator, downstream from the Process-L. The values of the parameters of this model are reported in Table 1 below.

**Table 1:** Model parameters.

Parameter	Value	Parameter	Value
*μ*	0.13 h^−1^	*q*	1/3
*k*	0.55 h^−1^	*p*	0.5
*α*_0_	0.05 h^−1^	*β*	0.0075 h^−1^
*G*	33.75	*I*_0_	9500 lux
*τ*_*x*_	24.2 h	*k*_*c*_	0.4 h

We define a 24 h- periodic light input

$$\begin{array}{c} I_{w}\left(t\right)=\{\begin{array}{cc} I_{w,\max}, & 0\leq t<16,\\ 0, & 16\leq t<24, \end{array} \end{array}$$ (7)

simply simulating the natural light-dark cycle, which is treated as the reference light in the following entrainment processes. The reference state trajectories in this model, denoted as $\bar{x}\left(t\right)$, ${\bar{x}}_{c}\left(t\right)$ and $\bar{n}\left(t\right)$, respectively, are the stable limit cycle solution of the JFK model with the periodic input *I*_*w*_(*t*). The time-optimal entrainment (TOE) problem is formulated as finding the light input *I*(*t*) that drives a given initial state to this periodic solution as quickly as possible. The jet-lag caused by rapid transmeridian travel is considered in this paper. We basically assume that the circadian rhythm of the entraining subject keeps synchronization with the local time in the starting point during the travel time, formally:

### PROBLEM (TOE)

Given the system dynamics (1)–(6) and initial conditions

$$\begin{array}{c} \left[x,x_{c},n\right]\left(0\right)=\left[\bar{x},{\bar{x}}_{c},\bar{n}\right]\left(T_{lag}\right), \end{array}$$ (8)

where *T*_*lag*_ ∈ (0, 24) is the amount of jet-lag, i.e., time shift between the destination and starting point of travel. We want to minimize *T* such that

$$\begin{array}{c} {∥[\begin{array}{c} x(T)\\ x_{c}\left(T\right) \end{array}]-[\begin{array}{c} \bar{x}\left(T\right)\\ {\bar{x}}_{c}\left(T\right) \end{array}]∥}^{2}-tol_{1}=0, \end{array}$$ (9)

using *I*(*t*) as the optimization variable, where *tol*_1_ = 0.01 corresponds to about 30 minutes difference in the circadian phase and is small enough to ignore. We assume that, when the entraining state trajectory reaches the reference one (achieves the stopping criterion), the reference light in (7) is applied on the subject. Thus, the entrainment light strategy in this model during the whole time is given as

$$\begin{array}{c} \hat{I}\left(t\right)=\{\begin{array}{cc} I^{*}\left(t\right), & t\leq T,\\ I_{w}\left(t\right), & t>T, \end{array} \end{array}$$

where I*(*t*) represents the optimal light input of the TOE problem. As $\left[\bar{x},{\bar{x}}_{c}\right]$ is the stable periodic solution of the dynamics with the input *I*_*w*_, the reference light *I*_*w*_ will drive the entrainment state [*x*, *x*_*c*_] to the reference state $\left[\bar{x},{\bar{x}}_{c}\right]$ gradually if they are close with each other. Note that *n* is introduced in the Process-L of the JFK model to simulate the nonlinear relation of the light input and circadian drive received by the SCN. It has a very fast time scale and very small effects on the stability of the limit cycle, which is shown in the following section. The full order model behaves just like the 2nd-order one. Therefore, *n* is ignored in the terminal condition (9). We also impose the constraint

$$\begin{array}{c} 0\leq I\left(t\right)\leq I_{\max}, \end{array}$$ (10)

for all *t* ∈ [0, *T*], where *I*_max_ is a parameter representing the maximum light intensity that can be used.

Throughout the paper, we define the **circadian phase**(in radian) of any point on the limit cycle of the circadian oscillation [*x*, *x*_*c*_, *n*] as

$$\begin{array}{c} \theta ≜-\tan^{-1}(\frac{x_{c}}{x}),\;\theta \in \left[0,2\pi \right). \end{array}$$ (11)

## 3 Model simplification

To reduce the complexity of solving the optimization problem above, some simpler models have been introduced. The optimization problem was then reformulated for the simplified models. In this section, we briefly review the simplified models and compare their behaviors under the periodic light input in Eq (7).

### 3.1 Second-order model

In this model, we exploit the fact that the Process-L (3) has a much faster time scale than the circadian oscillator. We use quasi steady-state approximation to assume that the Process-L is always at its steady-state. This yields

$$\begin{array}{c} n=\frac{\alpha (I)}{\alpha (I)+\beta }=\frac{\alpha_{0}{\left(\frac{I}{I_{0}}\right)}^{p}}{\alpha_{0}{\left(\frac{I}{I_{0}}\right)}^{p}+\beta }. \end{array}$$ (12)

Further, from (5) we have

$$\begin{array}{c} u=\frac{G\beta \alpha_{0}{\left(\frac{I}{I_{0}}\right)}^{p}}{\alpha_{0}{\left(\frac{I}{I_{0}}\right)}^{p}+\beta }. \end{array}$$ (13)

Since *u* is related to *I* through a static nonlinear mapping (13), we can simplify the circadian oscillation model by assuming that *u* is the control input and removing the Process-L from the model. The resulting model is therefore

$$\frac{dx}{dt}=\frac{\pi }{12}[x_{c}+\mu (\frac{1}{3}x+\frac{4}{3}x^{3}-\frac{256}{105}x^{7})+B],$$ (14)

$$\frac{dx_{c}}{dt}=\frac{\pi }{12}[qx_{c}B-{\left(\frac{24}{0.99729\tau_{x}}\right)}^{2}x-kxB],$$ (15)

$$B=\left(1-0.4x\right)\left(1-k_{c}x_{c}\right)u.$$ (16)

Further, we map the periodic input in (7) through (13) and obtain a 24 h- periodic circadian input

$$\begin{array}{c} U_{w}\left(t\right)=\{\begin{array}{cc} U_{w,\max}, & 0\leq t<16,\\ 0, & 16\leq t<24. \end{array} \end{array}$$ (17)

The reference state trajectories in this part, denoted as $\tilde{x}\left(t\right)$ and ${\tilde{x}}_{c}\left(t\right)$, respectively, are the stable limit cycle solution of the 2nd-order model with the periodic input *U*_*w*_(*t*).

For the 2nd-order model, we can define a surrogate for the time optimal entrainment problem (TOE) as follows:

#### PROBLEM (TOE-2nd Order)

Given the system dynamics (14)–(16) and initial conditions

$$\begin{array}{c} \left[x,x_{c}\right]\left(0\right)=\left[\tilde{x}\left(T_{lag}\right),{\tilde{x}}_{c}\left(T_{lag}\right)\right], \end{array}$$ (18)

minimize *T* such that

$$\begin{array}{c} {∥[\begin{array}{c} x(T)\\ x_{c}\left(T\right) \end{array}]-[\begin{array}{c} \tilde{x}\left(T\right)\\ {\tilde{x}}_{c}\left(T\right) \end{array}]∥}^{2}-tol_{1}=0, \end{array}$$ (19)

using *u*(*t*) as the optimization variable. We also impose the constraint

$$\begin{array}{c} 0\leq u\left(t\right)\leq u_{\max}, \end{array}$$ (20)

for all *t* ∈ [0, *T*], where *u*_max_ is a parameter representing the maximum circadian input that can be used.

### 3.2 First-order model

Exploiting the fact that the 2nd-order circadian model has a stable limit cycle, we can further reduce the 2nd-order model to one that basically only captures the dynamics of the *phase* of the oscillation, and thus ignores the *amplitude* of the oscillation. The first-order model is of the form

$$\begin{array}{c} \frac{d\theta }{dt}=\omega_{0}+f\left(\theta \right)u, \end{array}$$ (21)

where *θ* (in radians) is the circadian phase. The parameter *ω*_0_ is the so-called *free running frequency*, whose value *ω*_0_ = 2*π*/24.2 rad/h is chosen to match the frequency of the limit cycle (with *u*(*t*) ≡ 0) in the second-order model (14)–(16). The input *u* is the circadian input with the same interpretation as in (13). The function *f*(*θ*) is called the *phase response function* (or *phase response curve*) in the literature [16, 20, 23, 24]. Based on (21), we can interpret this function as a map from the timing of the introduction of an impulsive input to the resulting shift in the circadian phase.

We compute the phase response function *f*(*θ*) with the following procedure.

**Fig 1:**
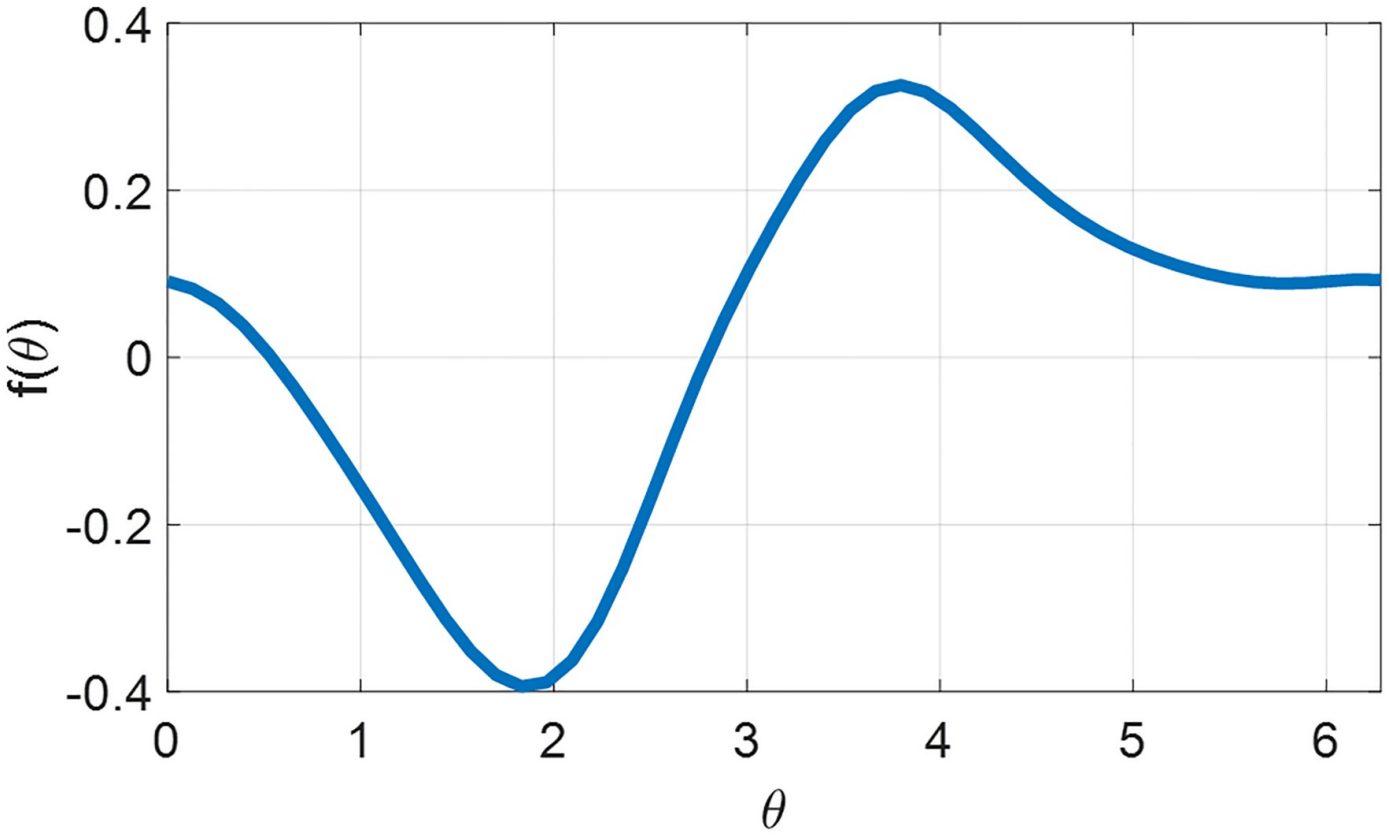
Phase response curve generated from the 2nd-order model.

Step 1 Obtain the free running periodic solution to (14)–(16) by setting *u*(*t*) ≡ 0. Denote this solution as $\left({\tilde{x}}_{f}\left(t\right),{\tilde{x}}_{cf}\left(t\right)\right)$ and the period as *T*_*ω*_. We assume the timing convention occurs at ${\tilde{x}}_{fc}\left(0\right)=0$ and ${\tilde{x}}_{f}\left(0\right)$ is at maximum.
Step 2 Choose different values of *τ* ∈ [0, *T*_*ω*_], run the model in (14)–(16) with initial conditions $x\left(0\right)={\tilde{x}}_{f}\left(\tau \right)$ and $x_{c}\left(0\right)={\tilde{x}}_{cf}\left(\tau \right)$ with the impulsive input signal
  $$\begin{array}{c} u_{\theta }\left(t\right)=\{\begin{array}{cc} u_{\max}, & t\in [0,\Delta ],\\ 0, & \text{else}. \end{array} \end{array}$$ (22)
  The corresponding impulsing phase is defined as
  $$\begin{array}{c} \theta =-\tan^{-1}(\frac{{\tilde{x}}_{cf}\left(\tau \right)}{{\tilde{x}}_{f}\left(\tau \right)}),\;\theta \in \left[0,2\pi \right), \end{array}$$ (23)
  where *u*_max_ = 0.2208 corresponds to *I*_max_ = 10000 lux. Here, Δ is a much shorter time interval than 24 hours. In our implementation, we choose Δ = 0.5 hour.
Step 3 Since (*x*, *x*_*c*_) will converge back to the free running periodic orbit, we define the resulting time shift *T*_*θ*_ (modulo the period *T*_*ω*_) such that
  $$\begin{array}{c} \lim_{t\to \infty }∥[\begin{array}{c} x(t)\\ x_{c}\left(t\right) \end{array}]-[\begin{array}{c} {\tilde{x}}_{f}\left(t+\tau +T_{\theta }\right)\\ {\tilde{x}}_{cf}\left(t+\tau +T_{\theta }\right) \end{array}]∥=0. \end{array}$$ (24)
  Then, the phase response function is given by
  $$\begin{array}{c} f\left(\theta \right)=\frac{2\pi T_{\theta }}{T_{\omega }u_{\max}\Delta }. \end{array}$$ (25)
  The resulting PRC is demonstrated as Fig 1, which is used for the formulation of the 1st-order model in (21).

Forcing the first-order model in Eq (21) with the periodic circadian input *U*_*w*_(*t*) from Eq (17) results in a periodic limit cycle (modulo 2*π*), where the circadian phase trajectory is denoted as $\bar{\theta }\left(t\right)$. For the first-order model, we can define a surrogate for the time-optimal entrainment problem (TOE) as follows:

#### PROBLEM (TOE-1st order)

Given the system dynamics (21) with an initial condition

$$\begin{array}{c} \theta \left(0\right)=\bar{\theta }\left(T_{lag}\right), \end{array}$$ (26)

minimize *T* such that

$$\begin{array}{c} |\theta \left(T\right)-\bar{\theta }\left(T\right)|-tol_{2}=0\;\text{mod}\;2\pi =0, \end{array}$$ (27)

using *u*(*t*) as the optimization variable, where *tol*_2_ = 0.1 also corresponds to about 30 minutes difference in the circadian phase. We also impose the constraint

$$\begin{array}{c} 0\leq u\left(t\right)\leq u_{\max}, \end{array}$$ (28)

for all *t* ∈ [0, *T*], where *u*_max_ is a parameter representing the maximum circadian input that can be used.

### 3.3 Behaviors under periodic light inputs

**Fig 2:**
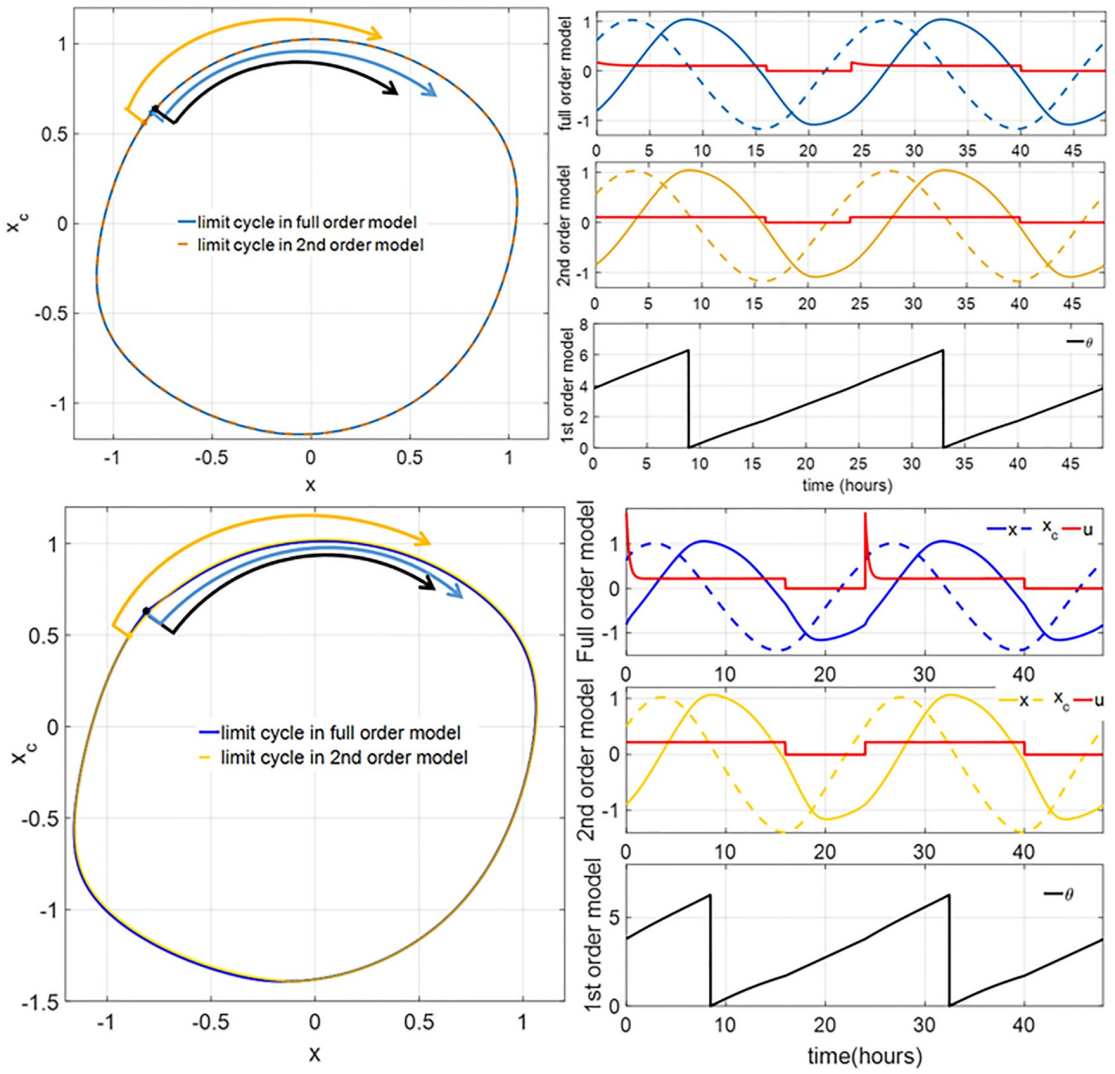
(Left column) Limit cycles of the full order and 2nd-order models corresponding to the circadian light intensities *I*_*w*,max_ of 100 lux (top) and 10000 lux (bottom). The blue and yellow arrows indicate the values of *x* and *x*_*c*_ at sunrise. The black arrow indicates the phase of the 1st-order model at sunrise. (Right column, first and second panels) The graphs of *x* (solid) and *x*_*c*_ (dash) of the limit cycles versus time for the full order and second-order systems, respectively, for *I*_*w*,max_ = 100 lux. (Right column, third panel) The graph of the circadian phase *θ* of limit cycle versus time for the first-order model for *I*_*w*,max_ = 100 lux. (Right column, fourth to sixth panels) The same as the first three panels for *I*_*w*,max_ = 10000 lux. The red curves represent the circadian drive input of each model. Note that the circadian drives of the 1st-order model in the third and sixth panels are equal to those in the second and fifth panels, respectively.

Under the periodic inputs *I*_*w*_(*t*) (for the full order model) and *U*_*w*_(*t*) (for the reduced models), the system exhibits stable limit cycles. Fig 2 shows a comparison between the periodic orbits of these models for *I*_*w*,max_ = 100 lux (comparable to the light intensity in a dim indoor space such as office corridors or elevators [25]), and *I*_*w*,max_ = 10000 lux (comparable to the light intensity outdoor on a bright day). The corresponding values of *U*_*w*,max_ (through (13)) are 0.1028 and 0.2208, respectively. We can make the following observations from these simulation data:

- The periodic orbits of the full order model and the second-order model are practically the same.
- There is a relative phase shift between these models, marked by the circadian phases at sunrise. At *I*_*w*,max_ = 100 lux, the largest gap is 0.0974 rad (22.31 min) between the first-order and second-order models. At *I*_*w*,max_ = 10000 lux, the largest gap is 0.1534 rad (35.15 min) between the second-order and the full order model.
- The impact of the Process-L is more significant at the higher circadian light intensity. This can be observed by comparing the waveforms of the circadian input *u*(*t*) in the full order and second-order models. At *I*_*w*,max_ = 10000 lux, *u*(*t*) has more pronounced spikes when the light is switched on, compared to at *I*_*w*,max_ = 100 lux.

## 4 Solution strategies for the time optimal entrainment problems

Solution strategies for TOE-2nd order and TOE-1st order have been reported in [19, 20], respectively. For completeness, we summarize the strategies in this section.

First, note that all system models are single input affine nonlinear systems. That is, they can be written in the following format

$$\begin{array}{c} \frac{d\xi }{dt}=\phi \left(\xi \right)+\gamma \left(\xi \right)\upsilon , \end{array}$$ (29)

where $\xi \in R^{n}$ is the system state, and υ∈R is the control input. We assume that the control input is bounded,

$$\begin{array}{c} 0\leq \upsilon \leq \upsilon_{\max}. \end{array}$$ (30)

Further, the time optimal entrainment (TOE) problem can be cast as a time optimal control problem with terminal state constraint, which can be formulated as minimizing the objective

$$\begin{array}{c} J=\int_{0}^{T}1\;dt, \end{array}$$ (31)

subject to the initial and terminal state constraints

$$\xi (0)=\xi_{\text{init}},$$ (32)

η(ξ(T),T)=0. (33)

The function $\eta :R^{n}\times R\to R$ can be used to represent the fact that the terminal state must be at a time-varying target state (i.e., the reference). In terms of the entrainment problem of the three models mentioned in this paper, we define the expression of *η* = 0 based on (9), (19) and (27), respectively.

Following the Pontryagin’s Maximum Principle, we formulate the augmented cost function

$$\begin{array}{c} L\left(\xi ,\upsilon ,T,\lambda ,\kappa \right)≜T+\int_{0}^{T}\lambda^{T}\left(t\right)(\phi \left(\xi \left(t\right)\right)+\gamma \left(\xi \left(t\right)\right)\upsilon \left(t\right)-\frac{d\xi }{dt})\;dt+\kappa^{T}\eta \left(\xi \left(T\right),T\right), \end{array}$$ (34)

where $\lambda \left(t\right)\in R^{n}$ is the co-state and *κ* is the Lagrange multiplier corresponding to the terminal state constraint.

The first variation of the objective *J* with respect to the input signal *υ*(*t*) is given by

$$\begin{array}{c} \delta J=\int_{0}^{T}\lambda^{T}\left(t\right)\gamma \left(\xi \left(t\right)\right)\delta \upsilon \left(t\right)\;dt, \end{array}$$ (35)

where *δυ*(*t*) is the perturbation of *υ*(*t*). The optimal control input can be found by

$$\begin{array}{c} \upsilon^{*}\left(t\right)=\arg\min_{\upsilon }\lambda^{*}{\left(t\right)}^{T}\gamma \left(\xi^{*}\left(t\right)\right)\upsilon , \end{array}$$ (36)

where ξ*(*t*) and λ*(*t*) are optimal state and co-state trajectories, respectively. We can see that the optimal control input therefore necessarily follows a bang-off strategy

$$\begin{array}{c} \upsilon^{*}\left(t\right)=\{\begin{array}{cc} 0, & \lambda^{*T}\left(t\right)\gamma \left(\xi^{*}\left(t\right)\right)>0,\\ \upsilon_{\max}, & \lambda^{*T}\left(t\right)\gamma \left(\xi^{*}\left(t\right)\right)<0. \end{array} \end{array}$$ (37)

**Remark 1** *The case where* λ*^T^ *γ*(ξ*) *is zero for a time interval of nonzero length is called a singular arc. We show in* [19] *that singular arcs do not affect the solution of TOE-2nd Order. Further, although not presented here, it is not difficult to see that the singular arc does not exist in the TOE-1st Order as it would violate the transversality condition explained below*.

The evolution of the state follows the model in (29), while the co-state evolves according to

$$\begin{array}{c} \frac{d\lambda^{*}}{dt}=-{\left(\frac{\partial \phi (\xi^{*})}{\partial \xi^{*}}+\frac{\partial \gamma \left(\xi^{*}\right)\upsilon^{*}}{\partial \xi^{*}}\right)}^{T}\lambda^{*}. \end{array}$$ (38)

Further, λ*(*t*) needs to satisfy its terminal condition,

$$\begin{array}{cc} {\left(\frac{\partial \eta (\xi^{*}\left(T^{*}\right),T^{*})}{\partial \xi }\right)}^{T}\kappa^{*} & =\lambda^{*}\left(T^{*}\right),\\ 1+\lambda^{*T}\left(T^{*}\right)(\phi \left(\xi^{*}\left(T^{*}\right)\right)+\gamma \left(\xi^{*}\left(T^{*}\right)\right)\upsilon^{*}\left(T^{*}\right))+\kappa^{*T}\frac{\partial \eta (\xi^{*}\left(T^{*}\right),T^{*})}{\partial T} & =0. \end{array}$$ (39)

which is also known as the *transversality condition* [26, 27]. Here, T* and κ* denote the optimal time and Lagrange multiplier, respectively.

To solve the optimal control problem above, we use two algorithms, which are explained below along with their strengths and weaknesses.

### 4.1 Direct shooting algorithm (DSA)

In this algorithm, we view the two point boundary value problem above as finding the appropriate initial co-state λ*(0). Observe that if (ξ*(*t*), υ*(*t*), λ*(*t*)) satisfy (29), (37), and (38), then so do (ξ*(*t*), υ*(*t*), *c*λ*(*t*)) for any positive scalar *c*. The right scalar *c* has to be chosen to satisfy the transversality condition (39). Therefore, the search space for λ*(0) can be reduced from $R^{n}$ to the unit sphere in $R^{n}$. It turns out that for TOE-2nd Order and TOE-1st Order, we can solve the two point boundary value problem rather efficiently. For TOE-2nd Order, this means λ*(0) can be sought on a circle. For TOE-1st Order, there are only two candidates for λ*(0). The basic direct shooting algorithm can then be expressed as follows:

Step 1 Create *N* sample points on the unit sphere in $R^{n}$. Denote them as λ_1_, ⋯, λ_*N*_.
Step 2 For each of *i* ∈ {1, ⋯, *N*} do: simulate *ξ*(*t*) and λ(*t*) forward using (29), (37), and (38), under initial conditions *ξ*(0) = *ξ*_init_ and λ(0) = λ_*i*_. Terminate the simulation when the final state constraint is satisfied,
  $$\begin{array}{c} t=T_{i},\;\eta \left(\xi \left(T_{i}\right),T_{i}\right)=0, \end{array}$$ (40)
  or when *t* reaches an upper bound *T*_max_. The upper bound *T*_max_ can be initially set based on the time needed for open-loop entrainment, and subsequently reduced as shorter entrainment times are found.
Step 3 Several locally optimal solutions can be found using the direct shooting method by searching abundant guesses of the initial co-state values. The optimal solution with the minimum entrainment time among all found solutions is treated as the globally optimal one, i.e., the optimal entrainment time is T* = min{*T*_*i*_}_*i*=1,⋯,*N*_.

Note that (29), (37), (38), and (39) provide us with a necessary condition for **local optimality**. This direct shooting algorithm provides us with a means to search (through sampling) for all solutions that satisfy this condition and therefore has the advantage of not getting trapped in local minima. The weakness of this algorithm is that for higher-order systems, such as the full order model, the number of samples generated can be impractically large. Another weakness is the co-state dynamics in (38) is unstable for the second-order and the full order models. This means forward numerical integration of this dynamics is unreliable for long periods of time, e.g., in jet-lag cases with long entrainment time.

**Fig 3:**
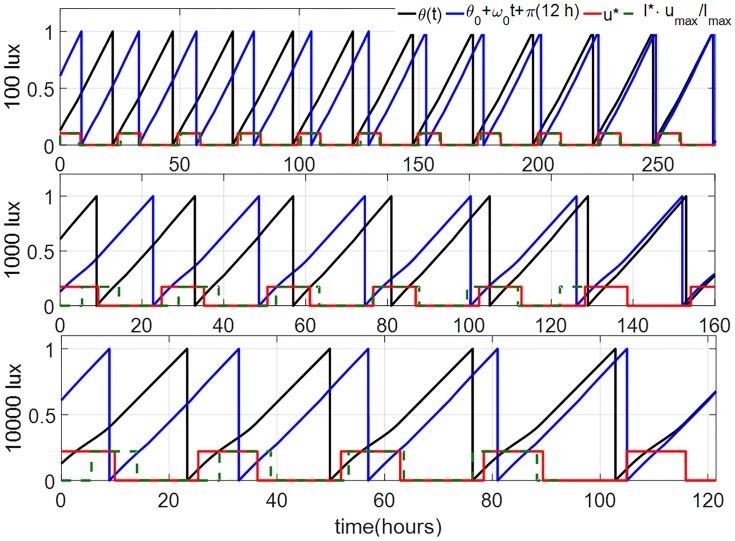
Solutions of TOE-1st order for the 12-hour jetlag case for the circadian lighting levels of 100 lux (top), 1000 lux (*u*_*max*_ = 0.1731) (middle) and 10000 lux (bottom), obtained using the DSA. The black and blue curves indicate the circadian phases of the subject and the reference, respectively. The red curve indicates the optimal light input of the 1st-order model. The green dash curves give the corresponding TOE solutions of the full order model.

**Fig 4:**
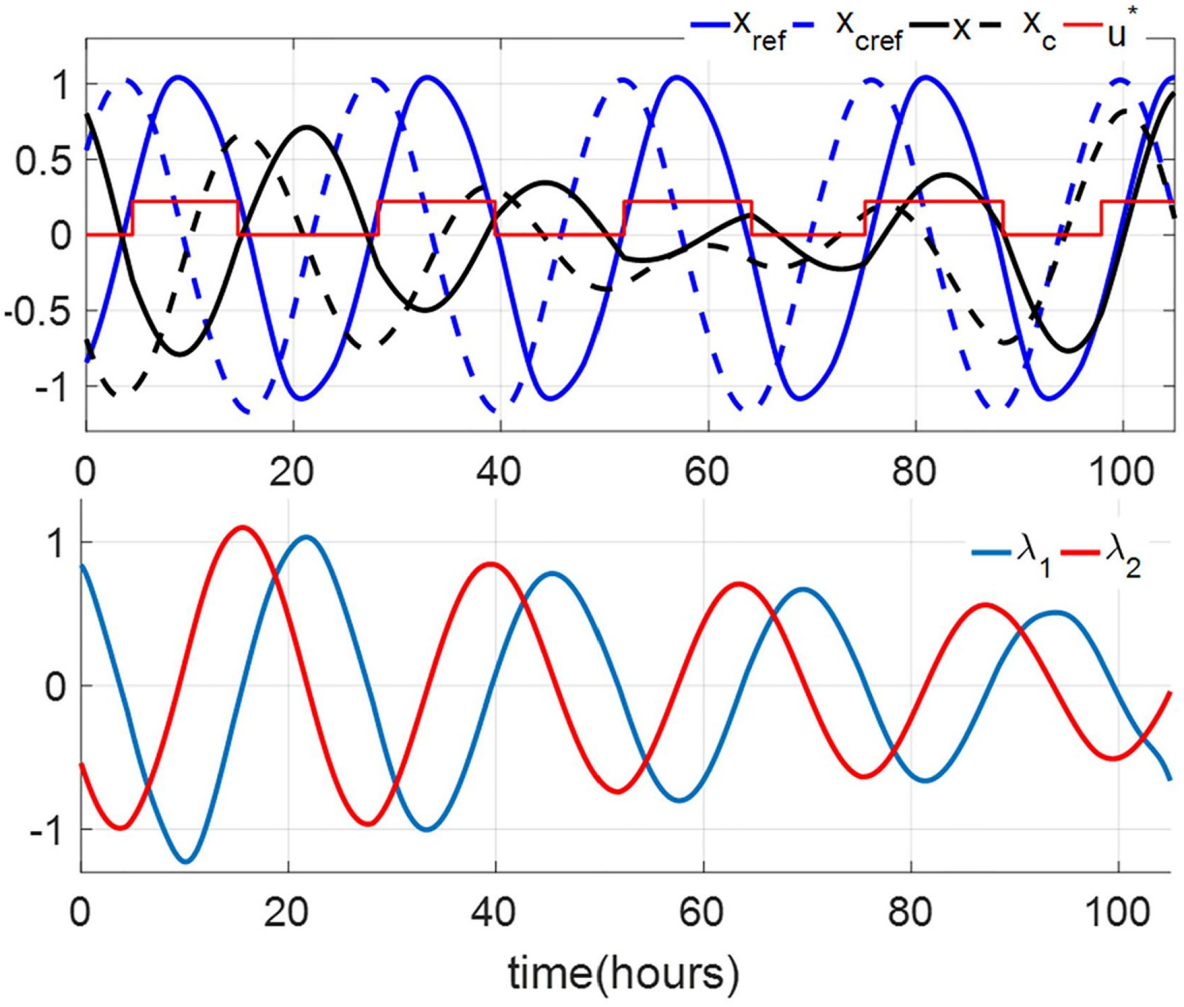
Solution of TOE-2nd Order for 12 hours jetlag case with circadian lighting level of 10000 lux, obtained using DSA. (Top) The black and blue curves indicate the circadian phases of the subject and the reference, respectively. The red curve indicates the optimal circadian input. (Bottom) The optimal co-state trajectories.

**Fig 5:**
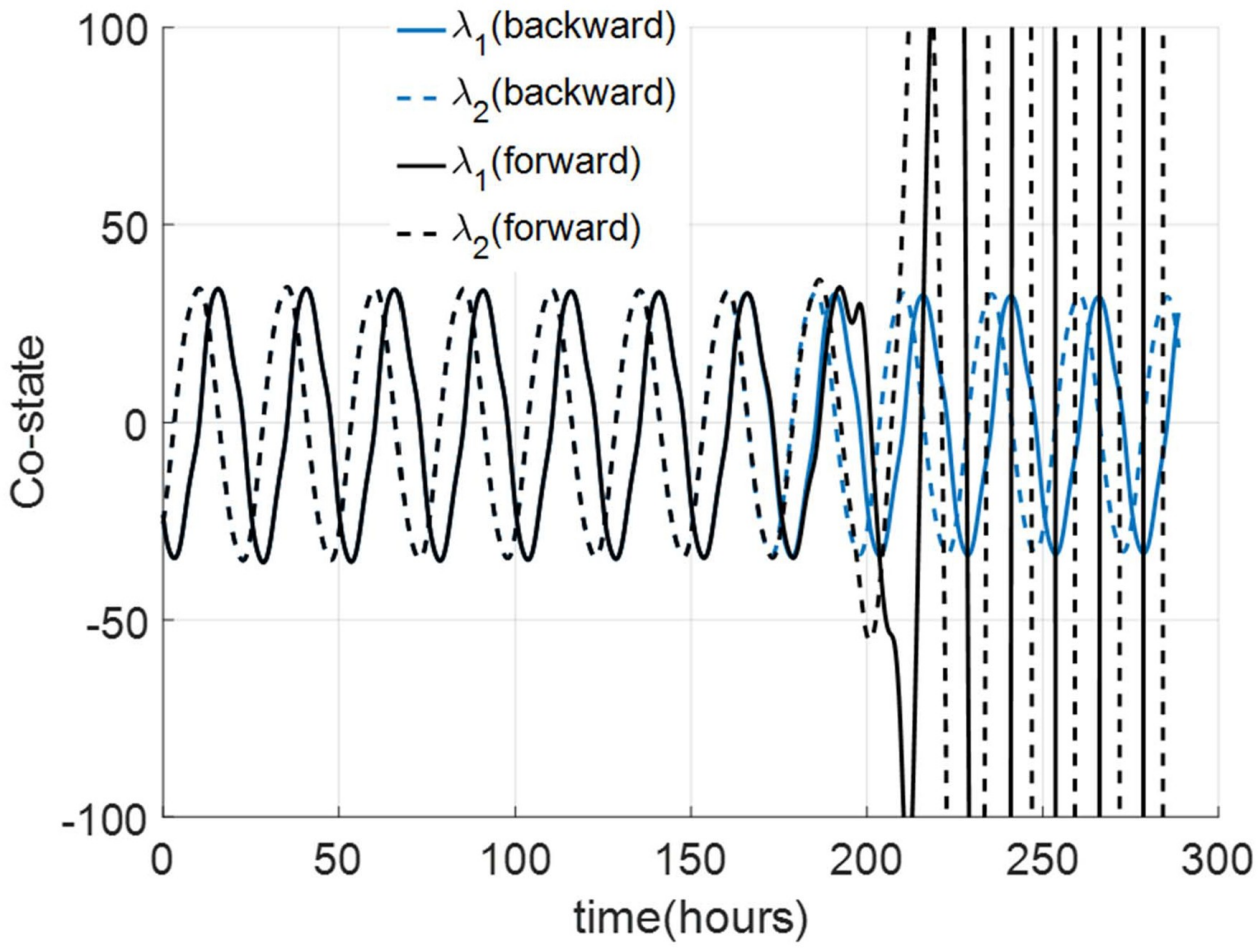
Numerical integrations of the co-state dynamics (38) in TOE-2nd Order. The backward integration (blue curves) is performed first. When forward integration is performed to re-trace the backward integration, the solutions (black curves) diverge.

Representatives of the optimization results using the DSA for a jet-lag case, where a subject whose initial circadian phase is at 6 am (corresponding to *θ*(0) = 0.8075 in TOE-1st Order) seeks to minimize her entrainment time for a 12-hour jet-lag are shown in Fig 3. We assume that *u*_max_ = 0.1028, *u*_max_ = 0.1731 and *u*_max_ = 0.2208 (corresponding to steady circadian lighting levels of 100 lux, 1000 lux and 10000 lux, respectively). Applying the same algorithm for the corresponding TOE-2nd Order case (corresponding to *x*(0) = 0.8033 and *x*_*c*_(0) = −0.6905) leads to a success for the bright circadian light cases (*u*_max_ = 0.2208 and 0.1731), as shown in Fig 4. For dim circadian light (*u*_max_ = 0.1028), the DSA does not converge because of the instability of numerical forward integration of the co-state dynamics in (38). This case is illustrated in Fig 5, where we can observe that even with the same initial co-state value at *t* = 0, λ_1_ and λ_2_ from the numerical forward integration diverge from the backward integration values at about 180 hours. As the optimal entrainment times with *I*_max_ = 1000 and 10000 lux are always less than 150 hours, the direct shooting algorithm has no issues in the forward integration in these cases.

### 4.2 Gradient descent algorithm (GDA)

This algorithm addresses the weaknesses of the DSA. The idea is to use gradient descent search for the optimal input υ*(*t*) by using the first variation in (35). The basic gradient descent algorithm can then be expressed as follows:

Step 1 Set *k* = 0. Choose an initial guess for control input $\upsilon_{0}:R_{+}\to \left[0,\upsilon_{\max}\right]$.
Step 2 Simulate *ξ*_*k*_(*t*) forward using (29), the initial condition *ξ*_*k*_(0) = *ξ*_init_, and the control input *υ*_*k*_(*t*) until *t* = *T*_*k*_ such that *η*(*ξ*_*k*_(*T*_*k*_), *T*_*k*_) = 0.
Step 3 Find the final condition for the co-state by solving
  $$\begin{array}{cc} {\left(\frac{\partial \eta (\xi_{k}\left(T_{k}\right),T_{k})}{\partial \xi }\right)}^{T}\kappa_{k} & =\lambda_{k}\left(T_{k}\right),\\ 1+\lambda_{k}^{T}\left(T_{k}\right)(\phi \left(\xi_{k}\left(T_{k}\right)\right)+\gamma \left(\xi_{k}\left(T_{k}\right)\right)\upsilon_{k}\left(T_{k}\right))+\kappa_{k}^{T}\frac{\partial \eta (\xi_{k}\left(T_{k}\right),T_{k})}{\partial T} & =0, \end{array}$$ (41)
  for *κ*_*k*_ and λ_*k*_(*T*_*k*_).
Step 4 Simulate λ_*k*_(*t*) backward using (38) and the final condition λ_*k*_(*T*_*k*_).
Step 5 Compute the descent direction using (35), i.e.,
  $$\begin{array}{c} \Delta_{k}\left(t\right)≜\lambda_{k}^{T}\left(t\right)\gamma \left(\xi_{k}\left(t\right)\right),\;t\in \left[0,T_{k}\right]. \end{array}$$ (42)
Step 6 Update the control input along the descent direction:
  $$\begin{array}{c} \upsilon_{k+1}\left(t\right)=\min\left(\max\left(0,\upsilon_{k}\left(t\right)-\alpha_{k}\Delta_{k}\left(t\right)\right),\upsilon_{\max}\right). \end{array}$$ (43)
  The step size *α*_*k*_ > 0 should be chosen such that the entrainment time is improved or the same as the previous value (*T*_*k*+1_ ≤ *T*_*k*_). In our simulations, we use a line search (bisection search, etc.) to solve the optimization problem given as follows:
  $$\alpha_{k}=\arg\min_{\alpha >0}T(\min\left(\max\left(0,\upsilon_{k}\left(t\right)-\alpha \Delta_{k}\left(t\right)\right),\upsilon_{\max}\right)).$$
  This process would be time-consuming, but it guarantees that the updating step decreases the entrainment time as much as possible or has no effects on the entrainment time in every iteration.
Step 7 Increment *k* by 1. Repeat from Step 2 until convergence. In our numerical simulations, we set the stopping criterion for the gradient descent process as
  $$\int_{0}^{T_{k+1}}\left|\upsilon_{k}\left(t\right)-\upsilon_{k+1}\left(t\right)\right|dt\leq 10^{-10}.$$
  The stopping criterion is used to verify that *υ*_*k*_(*t*) has reached or approximately reached the fixed point and the entrainment time cannot be improved. In most cases, the gradient descent algorithm takes at most 50 iterations to reach the stopping criterion mentioned above.

We can show that the following property characterizes fixed points of the iteration procedure above.

**Lemma 2** *The iteration reaches a fixed point, i.e., υ*_*k*+1_(*t*) = *υ*_*k*_(*t*) *for any positive step size α*_*k*_ *in Step 6 if and only if the following condition is satisfied for all t* ∈ [0, *T*_*k*_]

$$\begin{array}{c} \upsilon_{k}\left(t\right)=0\Rightarrow \Delta_{k}\left(t\right)\geq 0,\\ \upsilon_{k}\left(t\right)=\upsilon_{\max}\Rightarrow \Delta_{k}\left(t\right)\leq 0,\\ \upsilon_{k}\left(t\right)\in \left(0,\upsilon_{\max}\right)\Rightarrow \Delta_{k}\left(t\right)=0. \end{array}\}$$ (44)

**Proof:** Assume Eq (44) is satisfied. For all *t* ∈ [0, *T*_*k*_], when *υ*_*k*_(*t*) = 0, we have Δ_*k*_(*t*)≥0. Based on **Step 6**, for any positive *α*_*k*_, *υ*_*k*+1_(*t*) = *υ*_*k*_(*t*) = 0. It can also be proved that, for any *t* ∈ [0, *T*_*k*_], if *υ*_*k*_(*t*) = *υ*_max_, *υ*_*k*+1_(*t*) = *υ*_max_; if *υ*_*k*_(*t*) ∈ (0, *υ*_max_) and Δ_*k*_(*t*) = 0, we must have *υ*_*k*+1_(*t*) = *υ*_*k*_(*t*) for any *α*_*k*_ > 0. Therefore, the iteration reaches a fixed point. If Eq (44) is not satisfied, for example, if *υ*_*k*_(*t*) = 0 but Δ_*k*_(*t*) < 0 for any *t* ∈ [0, *T*_*k*_], we have *υ*_*k*+1_(*t*) > 0 by Eq (43). We can also prove that *υ*_*k*_(*t*) ≠ *υ*_*k*+1_(*t*) if the second or third row of Eq (44) does not hold. Therefore, *υ*_*k*_(*t*) is not a fixed point if it does not satisfy the condition in (44).

**Fig 6:**
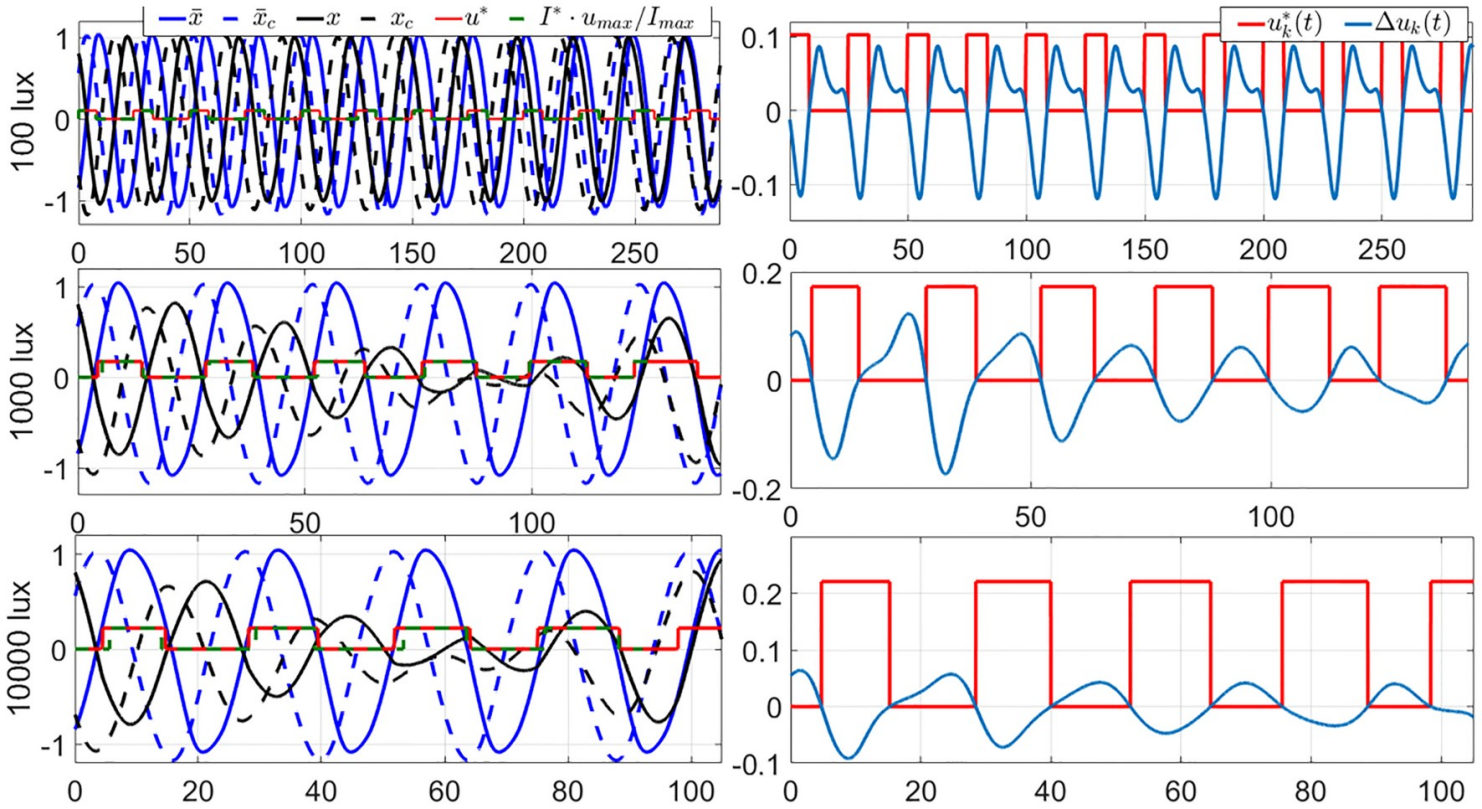
Locally optimal solutions of TOE-2nd Order for the 12-hour jet-lag case for the circadian lighting levels of 100 lux (*u*_*max*_ = 0.1028) (top row), 1000 lux (*u*_*max*_ = 0.1731) (middle row) and 10000 lux (*u*_*max*_ = 0.2208) (bottom row), obtained using the GDA. The left column are the graphs of the state trajectories, locally optimal light inputs, and the reference trajectories. The red curves indicate the optimal light inputs of the 2nd-order model. The green dash curves give the corresponding TOE solutions of the full order model. The right column are the graphs of the corresponding gradient of the entrainment time with respect to the circadian light input. Observe that, per Lemma 2, both solutions are indeed (locally) optimal.

Note that convergence of the iteration essentially means we hit a locally optimal control input. While the locality is a drawback of this algorithm, it has an advantage over the DSA because it does not require forward simulation of (38) that is numerically unstable. We demonstrate the use of GDA in solving the 12-hour jet-lag case for TOE-2nd Order (corresponding to *x*(0) = 0.8033 and *x*_*c*_(0) = −0.6905) under dim circadian light (*u*_max_ = 0.1028). We have shown in the previous section that DSA cannot be used to solve this problem. The TOE solution of this case is shown in the upper panels in Fig 6. Observe that since the co-states dynamics are integrated backward, we do not have the instability issue encountered in the DSA. Fig 6 also shows the TOE results of the 2nd-order model with *I*_max_ = 1000 and 10000 lux. We can observe in these cases that the amplitude suppression occurs in the oscillator of the core body temperature. The amplitude suppression regions are usually called the *phaseless set* [28], which, under higher light intensities, gives a ‘shortcut’ for the circadian states to follow and shortens the entrainment time. We can also observe in the amplitude suppression cases that, in the first two days, the light inputs are almost centered around the minimum value of *x*, i.e., the minimum value of the core body temperature. These light strategies are very similar to those in the trials in [29], whose results also prove that amplitude suppression contributes to the rapid circadian phase shift (as strong type 0 PRC) and fast entrainment under bright light.

**Fig 7:**
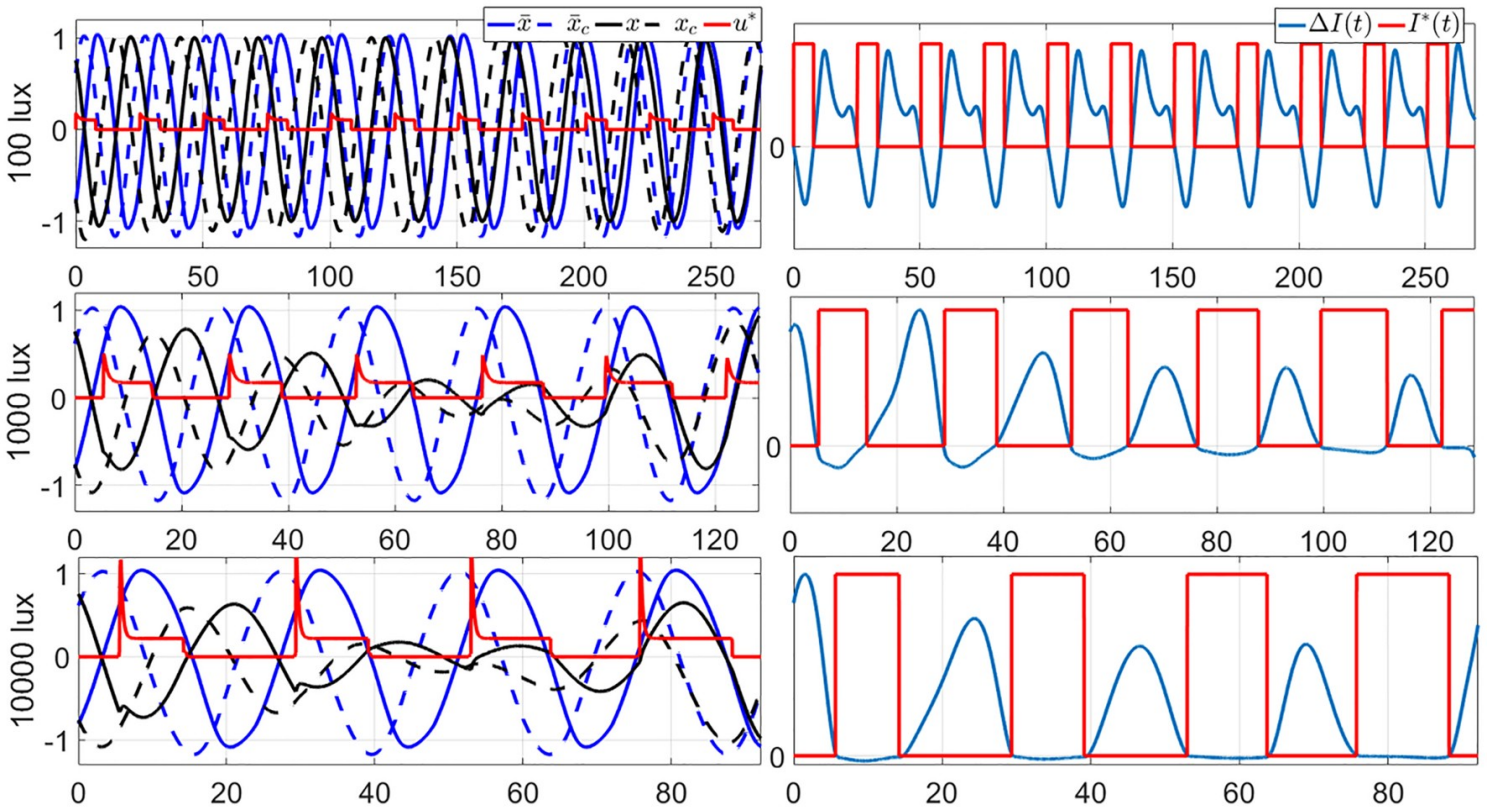
Locally optimal solutions of TOE-full order for the 12 hours jet-lag case for the circadian lighting levels of 100 lux (top row), 1000 lux (middle row) and 10000 lux (bottom row), obtained using GDA. On the left column are the graphs of the state trajectories, locally optimal light input, and the reference trajectories. The red curve indicates the optimal circadian input of the full order model. On the right column are the graphs of the corresponding gradients and light inputs. Observe that, per Lemma 2, both solutions are indeed (locally) optimal.

**Fig 8:**
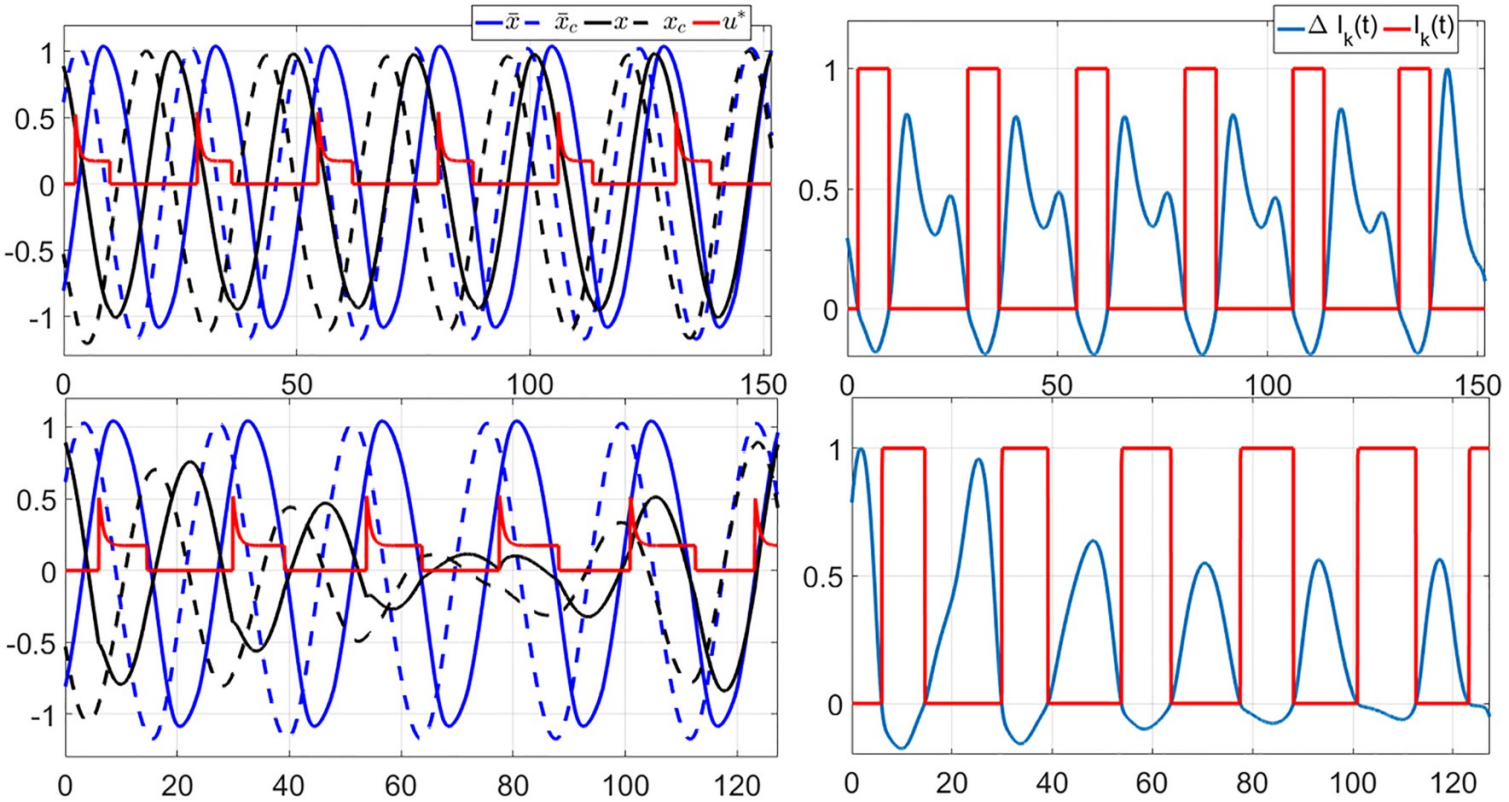
(Left) Two locally optimal solutions of TOE (full order) for the 11-hour jet-lag case with *I*_max_ = 1000 lux, obtained by using the GDA with two different initial guesses of the circadian light input signal. (Right) Graphs of the corresponding optimal circadian light inputs I*(*t*) and the descent direction Δ(*t*). We can also observe that, per Lemma 2, both solutions are (locally) optimal.

**Fig 9:**
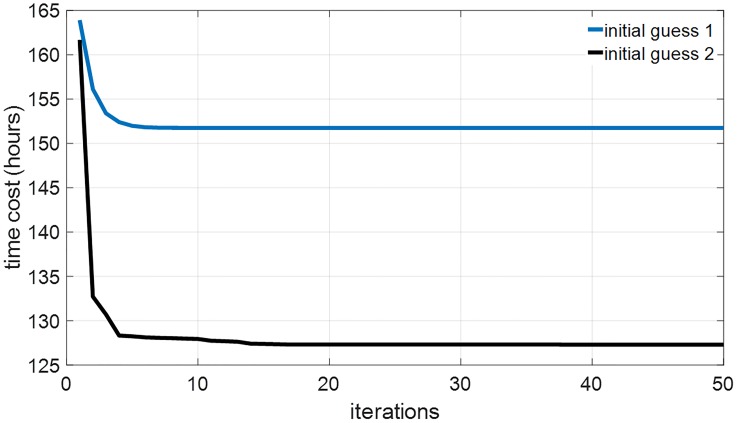
The optimization objective (i.e., entrainment time) improves over the iterations of the GDA for both of the solutions shown in Fig 8. The initial guess 1 in the blue curve in this figure converges to the TOE solution in the upper panels in Fig 8, the initial guess 2 in the black curve in this figure converges to the TOE solution in the lower panels in Fig 8.

We also apply GDA on the full order TOE problem. Again, we assume that the subject’s initial circadian phase is at 6 am (corresponding to *x*(0) = 0.7562, *x*_*c*_(0) = −0.7591, and *n*(0) = 0.4061). We consider 3 values of *I*_max_, 100 lux (dim indoor space), 1000 lux (overcast day outdoor or bright indoor space), and 10000 lux (full daylight outdoor). Fig 7 demonstrates the 12-hour minimum-time entrainment cases with *I*_max_ = 100, 1000 and 10000 lux and TOE solutions. We can also observe that the amplitude suppression occurs in the bright light cases, e.g. 1000 and 10000 lux, these results are consistent with those in the 2nd-order model. Fig 8 shows the results of applying the GDA on the 11-hour jet-lag case for the full order model, using *I*_max_ = 1000 lux. The fact that the locally optimal solutions provided by the GDA are generally not unique is demonstrated in Fig 8, where two solutions are shown to satisfy the conditions in Lemma 2. Fig 9 shows how the objective function improves over the iterations of the GDA for the two solutions shown in Fig 8.

### 4.3 Combining direct shooting and gradient descent algotihms

Local optimality of the results of GDA implies that their quality heavily depends on the initial guess of the optimal control input. On the other hand, DSA can be used effectively to find the global optimal solution for lower-order models. We then combine the use of both algorithms by using the solutions obtained using DSA on a lower-order model to warm start the GDA. Specifically, if u*(*t*) is the optimal circadian input found by applying DSA on a 1st- or 2nd-order model, then our initial guess for the optimal circadian light input intensity I*(*t*) for the full order model is determined by

$$\begin{array}{c} I^{*}\left(t\right)=\{\begin{array}{cc} 0, & u^{*}\left(t\right)=0,\\ I_{\max}, & u^{*}\left(t\right)=u_{\max}. \end{array} \end{array}$$ (45)

Then, we apply the GDA to further optimize I*(*t*). Note that we do not assume that the GDA will converge to I*(*t*) that only assumes two values, 0 or *I*_max_.

**Fig 10:**
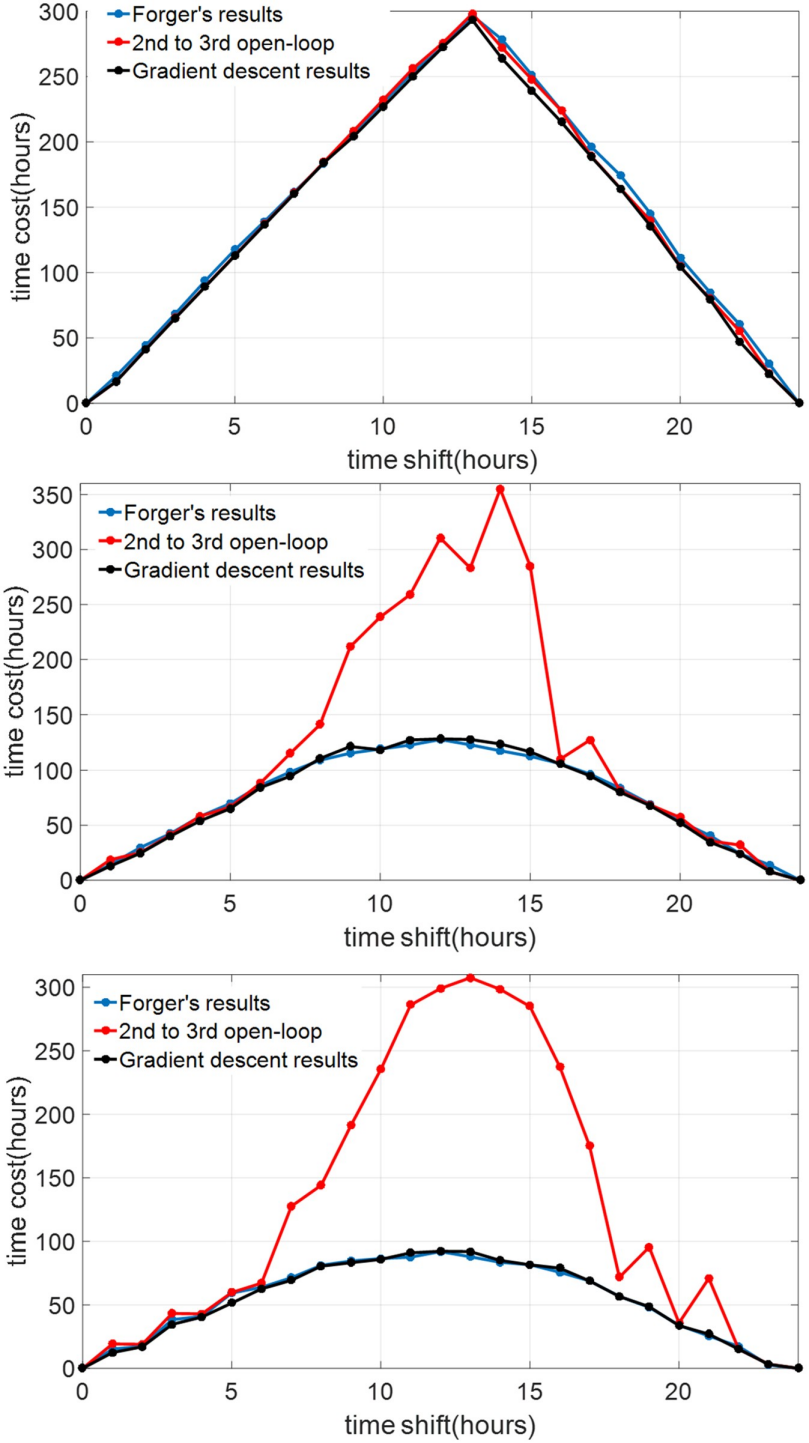
Comparison of the entrainment costs for the TOE (full order) problem for various jet-lag cases with the levels of circadian light intensity of 100 lux (top), 1000 lux (middle), and 10000 lux (bottom). The black curves represent results obtained using the GDA algorithm with initial guesses described in Sec. 4.3, while the blue curves represent those from [22]. The red curves represent the results of applying the optimal solutions of the corresponding TOE 2nd-order problems to the full order model. These are the initial guesses before the GDA is applied.

We compare the performance of the solutions that we obtained using this strategy with published results by Serkh and Forger [22]. The entrainment time for various jet-lag cases for our results and those from [22] are shown in Fig 10. In the same figure, we can also see the performance improvement between the solutions before the application of the GDA algorithm (thus, the initial guesses) and the solutions after the GDA algorithm. The comparison between the blue (results in [22]) and black curves (our results) verifies the performance of our algorithm. Also, the TOE light inputs from our algorithm (shown as the green dash curves in Fig 6) are also consistent with those in [22] (in particular, Fig S13 in [22]).

**Fig 11:**
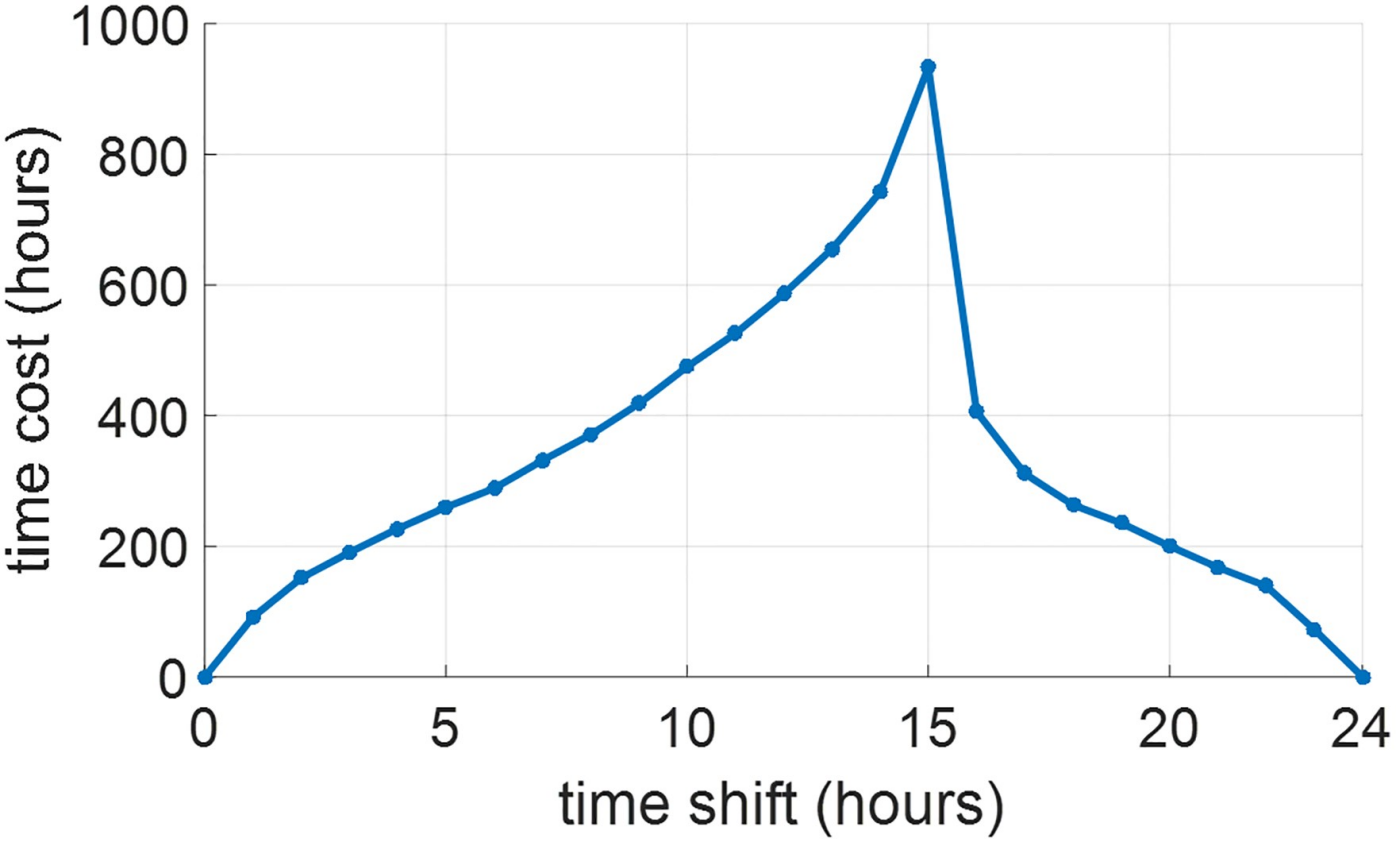
Entrainment time by the natural light-dark cycle in (7) with *I*_*w*,max_ = 100 lux.

The entrainment time in Fig 10 implies that the maximum entrainment time in our gradient descent results occurs at around 12-13 hours shift (westward travel), which means that the JFK model is almost east-west symmetrical. These results are consistent with those in [22], which addressed the minimum-time entrainment problem of the same circadian model. However, if we only use the natural light-dark cycle in (7) with *I*_*w*,max_ = 100 lux for entrainment without any other light input, the entrainment time in Fig 11 demonstrates that the JFK model is still east-west asymmetrical as maximum entrainment time occurs at around 15 hours westward shift. These results are consistent with those in [28], which also considered the entrainment of the JFK model using the natural light-dark cycle. These results imply that the JFK model is east-west asymmetrical under natural light but the optimal light inputs for minimum-time entrainment can remove this east-west asymmetry.

The solution strategy proposed by Serkh and Forger is briefly described as follows. It assumes that the optimal light input I*(*t*) only has two values, 0 or *I*_max_. This assumption is motivated by the fact that time optimal control problems with bounded input constraints generally have *bang-bang* or *bang-off* optimal solutions [26, 27]. The exceptions of this case are when the optimal solutions lie in a so-called *singular arc* [26, 27].

The switching time instants of the light input are parameterized as *t*_1_, *t*_2_, ⋯, *t*_*N*_ and considered as the optimization variables. The optimal control problem in TOE is reformulated as follows. To represent entrainment, the concept of isochrons is used. Essentially, this is established by using a *phase function* $\varphi :R\times R\to S^{1},$ such that two pair of states of the circadian oscillator (*x*_1_, *x*_*c*1_) and (*x*_2_, *x*_*c*2_) are considered to have the same phase if *φ*(*x*_1_, *x*_*c*1_) = *φ*(*x*_2_, *x*_*c*2_). Then, the following objective is optimized

$$\begin{array}{c} J=T+C\cdot {\left(∥[\begin{array}{c} x(T)\\ x_{c}\left(T\right) \end{array}]∥-∥[\begin{array}{c} \bar{x}\left(T\right)\\ {\bar{x}}_{c}\left(T\right) \end{array}]∥\right)}^{2}, \end{array}$$ (46)

under the constraint

$$\begin{array}{c} \varphi \left(x_{1}\left(T\right),x_{c1}\left(T\right)\right)=\varphi \left(x_{2}\left(T\right),x_{c2}\left(T\right)\right). \end{array}$$ (47)

The constant *C* > 0 in (46) represents the trade-off between minimizing the end time and matching the amplitude of the circadian oscillator with that of the reference signal. The optimal switching time instants $t_{1}^{*},t_{2}^{*},\ldots ,t_{N}^{*}$ are then computed using a gradient descent technique. Compared with [22], the algorithm used in this paper is proposed without the assumption that the minimum-time optimal input is always bang-off and computes the light input during the whole entrainment process instead of only the switching times. The results in [19] and [30] show the existence of singular arcs in the minimum-time entrainment solutions in several circadian rhythm models. These singular arcs violate the assumption that the optimal input is always bang-off. Therefore, our algorithm is more general in solving the optimal solution in various models.

## 5 The impacts of model reduction on the TOE solutions

In Sec. 3, we discussed two steps of model reduction of the full order JFK model. First, we ignored the dynamics of the Process-L and introduced the 2nd-order model. Second, we ignored the amplitude of the oscillation and introduced the 1st-order model. Here, we study the impacts of these model reduction steps on the solutions of the time-optimal entrainment problems. The TOE solutions of the full order model are also demonstrated in Figs 3 and 6. Comparing them with the TOE solutions of the 1st-order and 2nd-order models, we observe that, when the light input is dim (100 lux in the upper panels), the light on-off switching times in the TOE solutions of the 1st-order and 2nd-order models are both very similar with those in the full order model, while the difference between the 1st-order and full order model is enlarged when the light intensity is increased (middle and lower panels in Fig 3). The TOE solutions of the 2nd-order model are close to those of the full order model even under a bright light input, as demonstrated in the lower panel in Fig 6. However, even if we apply the optimal light input from the 2nd-order model on the full order model in the form of (45) in the bright light cases, it still brings a large difference in the entrainment time from the optimal entrainment time in many cases, shown as Fig 10. Specifically, for jet-lag cases, we evaluate the performance of the solutions derived using the lower-order models when applied on the higher-order models. For this, we simply replay the computed optimal control input trajectory u*(*t*) on the higher-order models until we reach entrainment, which is marked by the circadian states of the subject matching those of the reference, within a certain error tolerance band. If at the end of the computed u*(*t*) we do not reach entrainment, we append the control input with the reference input trajectory until we reach entrainment. As a concrete example, suppose that we consider the 12-hour jet-lag case with the maximum circadian lighting level of 10000 lux, and want to replay the optimal control input trajectory u*(*t*) computed using the 1st-order model on the full order model. The trajectory u*(*t*) in this case has a duration of 121 hours, as shown in Fig 3. The same 12-hour jet-lag case for the full order model is associated with the initial conditions

$$\left[x,x_{c},n\right]\left(0\right)=\left[0.7562,-0.7591,0.4061\right].$$

We then simulate the full order JFK model with the initial conditions above under the circadian light intensity signal I*(*t*) obtained using Eq (45) until entrainment is reached or *t* = 121 hours. If we do not reach entrainment by *t* = 121 hours, we continue I*(*t*) with the reference circadian lighting,

$$I^{*}\left(t\right)=I_{w}\left(t\right),\;t>121,$$

until entrainment is reached. Physically, this step means exposing the subject to the local/reference circadian lighting until his circadian rhythm is synchronized with the local/reference circadian rhythm.

**Fig 12:**
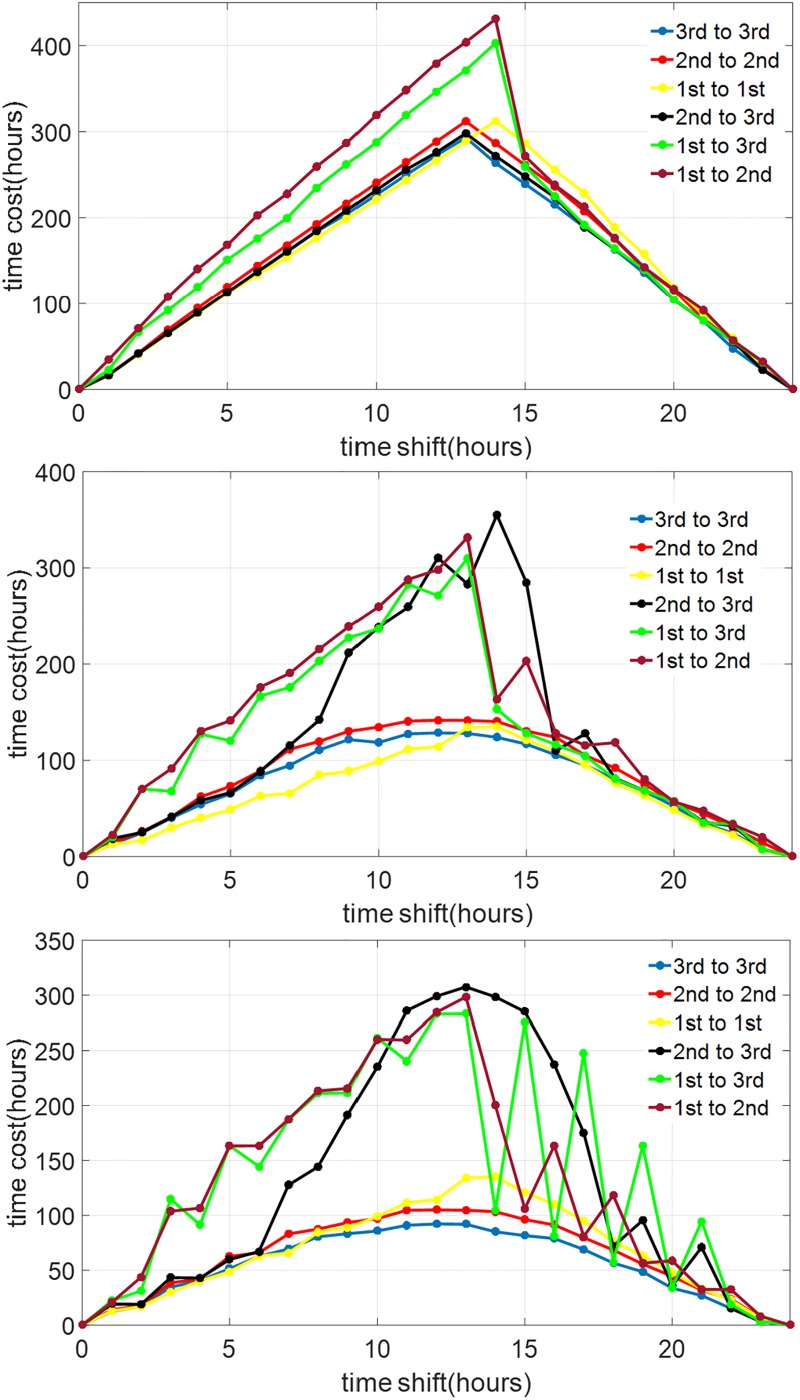
Entrainment time when the optimal input derived using a lower-order model is applied to the higher-order models. The top, middle, and bottom graphs represent the cases of circadian light intensity of 100 lux, 1000 lux, and 10000 lux, respectively. The graph labeled as “1st to 1st” represents the case when the result from the first-order model is applied to the first-order model, thus the optimal solution itself. The graph labeled as “1st to 3rd” represents the case when the result from the first-order model is applied to the full order model, and so on.

Fig 12 shows the results from the procedure above. We can observe that at lower circadian light intensity the gaps between the solutions of the time optimal entrainment problem of the reduced model and those of the full order model are smaller. In particular, at *I*_max_ = 100 lux, the solutions obtained using the second order are very similar to those from the full order model. However, the solutions obtained using the first order model already deviate quite significantly from those from the higher order models. This indicates that taking into account *the amplitude dynamics is relatively more important than the Process-L dynamics* in solving the time optimal entrainment problem.

## 6 Feedback implementation of the TOE solutions

The solution strategies obtained with all of the approaches described in the previous section are open loop in nature. Given an initial state and the reference state trajectory, the entire optimal control input trajectory υ*(*t*) and the corresponding optimal state trajectory ξ*(*t*) can be computed. However, because of Bellman’s Principle of Optimality [26, 27], we also know that the optimal input can be given in terms of an optimal feedback law υ*(ξ*, *t*). In this section, we discuss our approach to construct such an optimal feedback law from the obtained open loop optimal solutions.

### 6.1 Feedback implementation of the TOE solutions—First order case

A feedback implementation of the TOE 1st-order problems amounts to expressing the optimal circadian input u*(*t*) as a function of the subject’s circadian phase *θ*(*t*) and the reference circadian phase $\bar{\theta }\left(t\right)$. We have obtained and reported such feedback laws in our prior publication [20].

**Fig 13:**
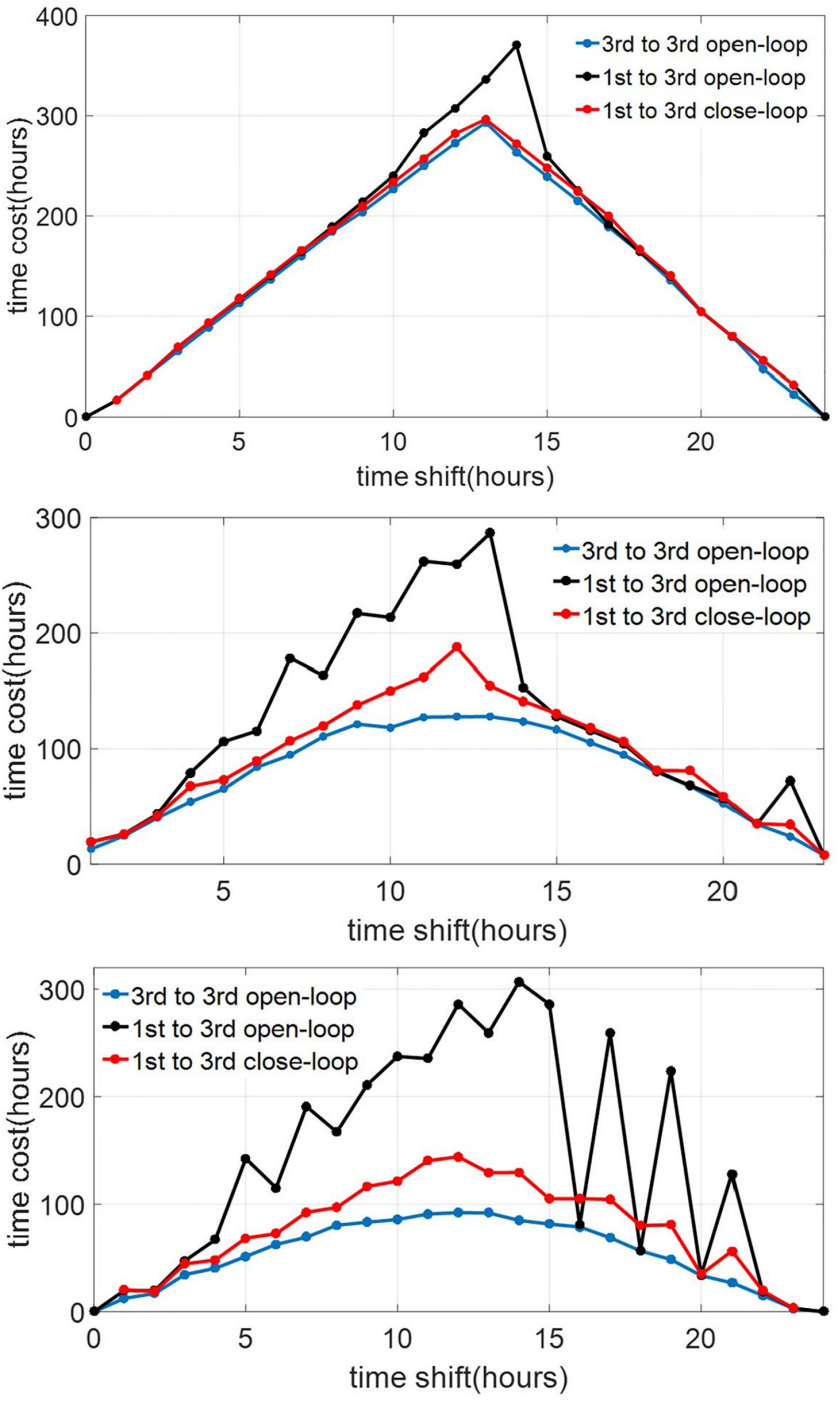
Entrainment time when the optimal input derived using the first-order model is applied to the full order models. The top, middle, and bottom graphs represent the cases of circadian light intensity of 100 lux, 1000 lux, and 10000 lux, respectively. The red and black curves represent the results from the feedback and open-loop implementations, respectively. The blue curves represent results from the full order TOE, which are shown as benchmarks.

Feedback implementation of the optimal control solutions confers more robustness to the solutions, e.g., under modeling error, compared to the open-loop implementation of the solutions. By open-loop implementation we mean simply replaying the optimal control input, as done in Sec. 5. We demonstrate this point by comparing the performance of the open-loop and feedback implementations of the solutions of TOE 1st-order on the full order model. The data are shown in Fig 13.

### 6.2 Feedback implementation of the TOE solutions—Full order case

Applying the idea above to the solution of full order TOE means we need to compute the optimal circadian light input I* as a function of the three states of the system (*x*, *x*_*c*_, and *n*) and the states of the reference system ($\bar{x}$, ${\bar{x}}_{c}$, and $\bar{n}$). However, since the reference trajectory is periodic, we can replace the states of the reference system with its circadian phase *θ*_*r*_, where the definition of the circadian phase is given in Eq (11). Further, we aim for the feedback law to be independent of *n* (the state of the Process L), because the implementation of such feedback law would require measuring (or estimating) *n*, which has much faster dynamics than the circadian rhythm.

The procedure for constructing the feedback law is as follows:

Step 1 For a given *I*_max_, collect a number of optimal trajectories $\left[x^{*}\left(t\right),x_{c}^{*}\left(t\right),n^{*}\left(t\right),\theta_{r}\left(t\right),I^{*}\left(t\right)\right]$ each with different initial conditions $\left[x^{*}\left(0\right),x_{c}^{*}\left(0\right),n^{*}\left(0\right)\right]$.
Step 2 Generate *N*_*s*_ data points by sampling the trajectories. That is, each data point $\left[x_{i}^{*},x_{c,i}^{*},n_{i}^{*},\theta_{r,i},I_{i}^{*}\right]\in R^{5}$, *i* ∈ {1, ⋯, *N*_*s*_} is a point on a trajectory described in Step 1.
Step 3 Find an interpolation function $F:R^{3}\to R$ that interpolates $\left[x_{i}^{*},x_{c,i}^{*},\theta_{r,i}\right]\to I_{i}^{*}$, *i* ∈ {1, ⋯, *N*_*s*_}. For the results reported in this paper we use a feedforward neural network trained by gradient propagation and cross-entropy loss function with a single hidden layer of 32 neurons (implemented by the patternnet function in Matlab) to represent the feedback function $F:R^{3}\to \left\{0,I_{\max}\right\}$.

**Fig 14:**
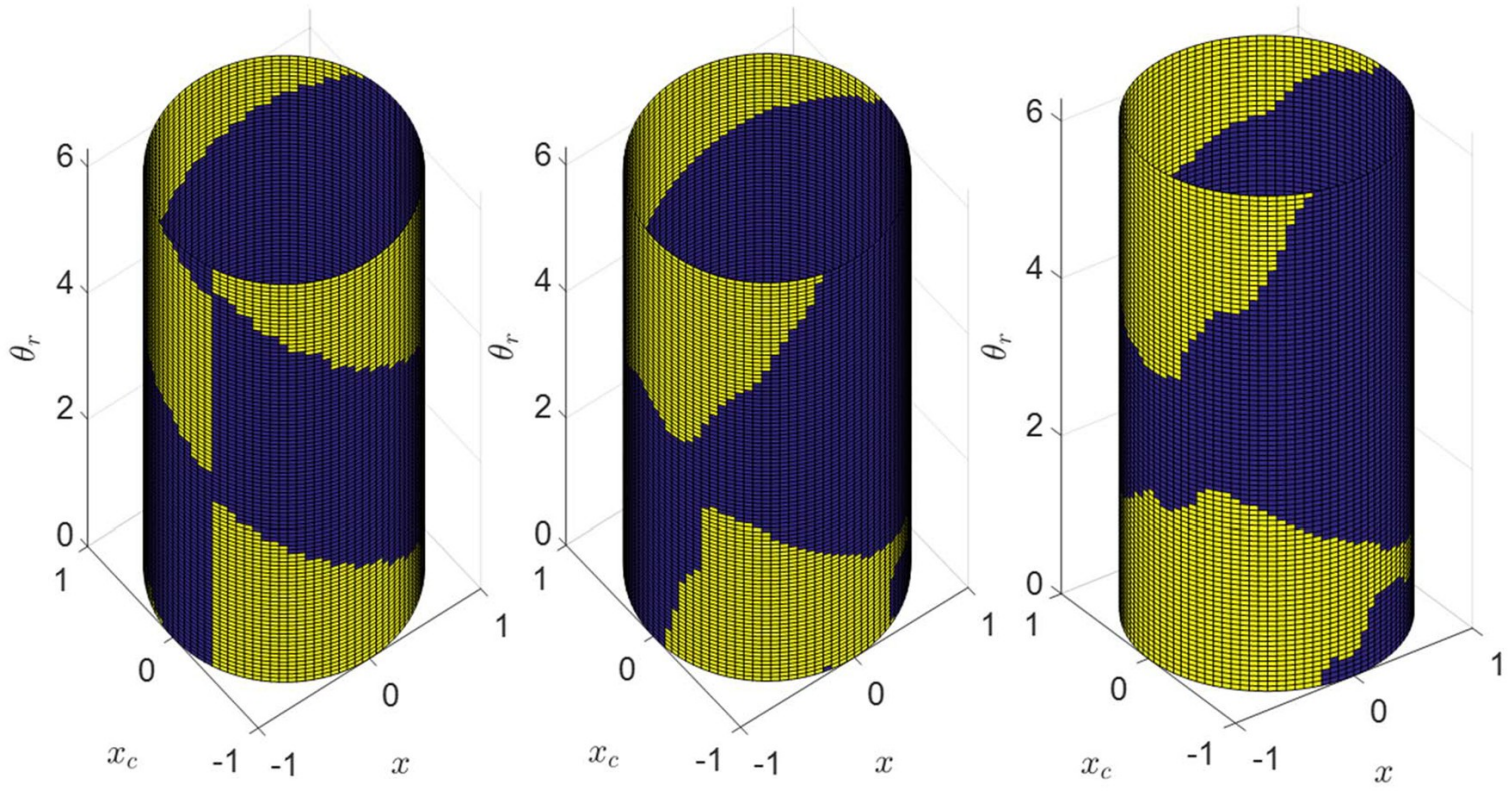
Optimal feedback control laws learned from the optimal feedforward solutions data for three levels of circadian light intensity, 100 lux (left), 1000 lux(center), and 10000 lux (right). Blue and yellow indicate cases where the optimal circadian light is maximum and zero, respectively.

Fig 14 shows the representations of the feedback controllers that we generated using this procedure for 3 values of *I*_max_, 100 lux (dim indoor space), 1000 lux (overcast day outdoor or bright indoor space), and 10000 lux (full daylight outdoor). The optimal trajectories for the training data are obtained by solving 23 cases of jetlag (*T*_*lag*_ ∈ {1, 2, ⋯, 23}) and sampled at intervals of 0.01 hour.

We evaluate the robustness of the feedback controller in three ways, as described below.

#### Cross-validation with new jetlag cases

**Fig 15:**
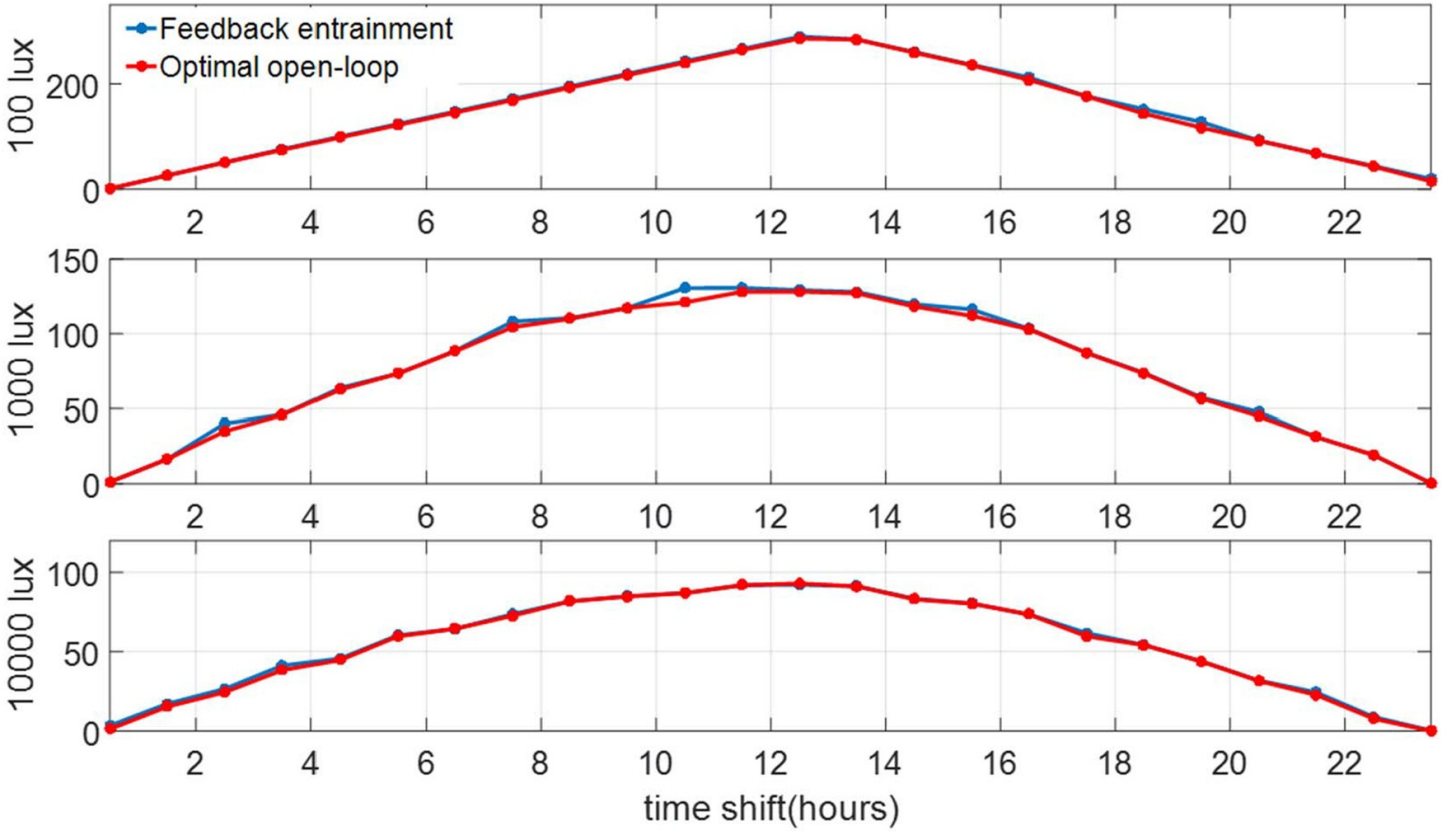
Performance of the feedback optimal control compared to that of the open-loop optimal solutions for jet-lag cases with $T_{lag}\in \left\{\frac{1}{2},1\frac{1}{2},\cdots ,23\frac{1}{2}\right\}$ for three levels of circadian light intensity. The vertical axis denote entrainment time in hours.

We apply the learned feedback controllers on jetlag cases that are not used in the training data set. Specifically, we consider the jetlag cases with $T_{lag}\in \left\{\frac{1}{2},1\frac{1}{2},\cdots ,23\frac{1}{2}\right\}$. We compare performance of the optimal open loop solutions of the TOE as described in Sec. 4.3 for these cases with the respective performance of the learned feedback controllers. The results are shown in Fig 15. We can confirm that the feedback controllers indeed perform well in this cross-validation.

#### Robustness w.r.t. *n*(0)

**Fig 16:**
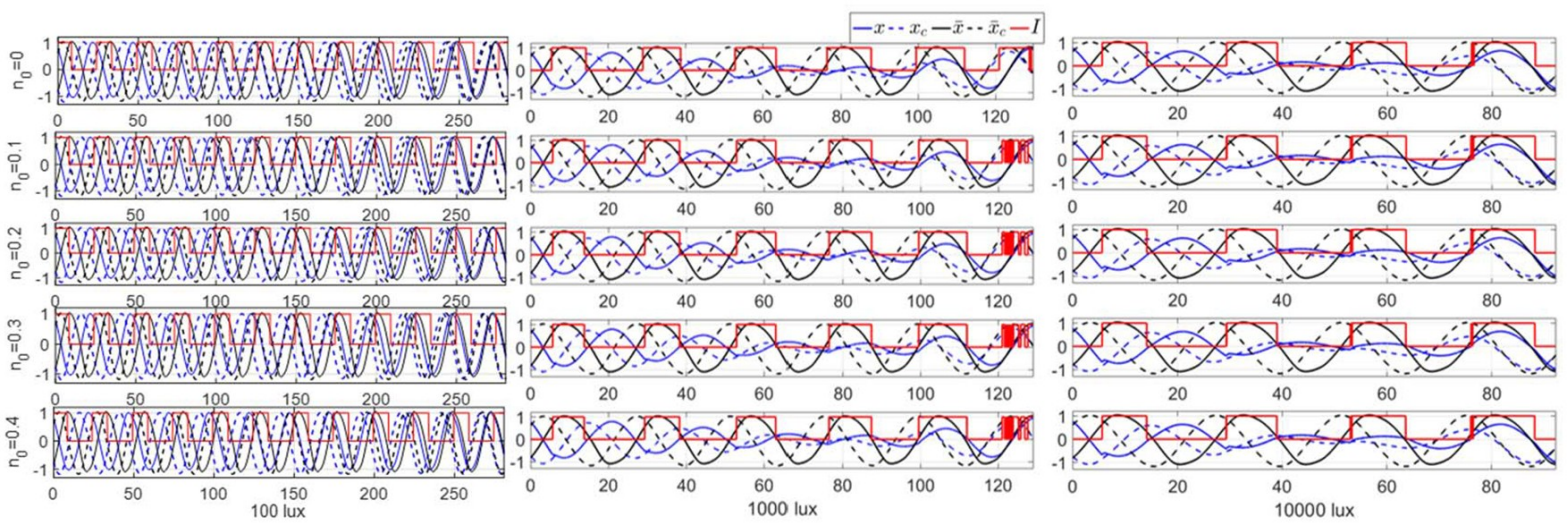
Performance of the feedback optimal control for the 12-hour jet-lag case with three levels of circadian light intensity and various initial conditions for *n*(0). Observe that *n*(0) (and consequently *n*(*t*)) only has little effect on the entrainment times.

The feedback controllers ignore the influence of *n*(*t*) in computing the optimal control response. To evaluate the validity of the assumption that *n*(*t*) can be ignored, we use the feedback controllers under various initial conditions *n*(0). The results shown in Fig 16 confirm that *n*(*t*) plays a very small role in the performance of the feedback controlled system.

#### Robustness w.r.t. change in *I*_max_

**Fig 17:**
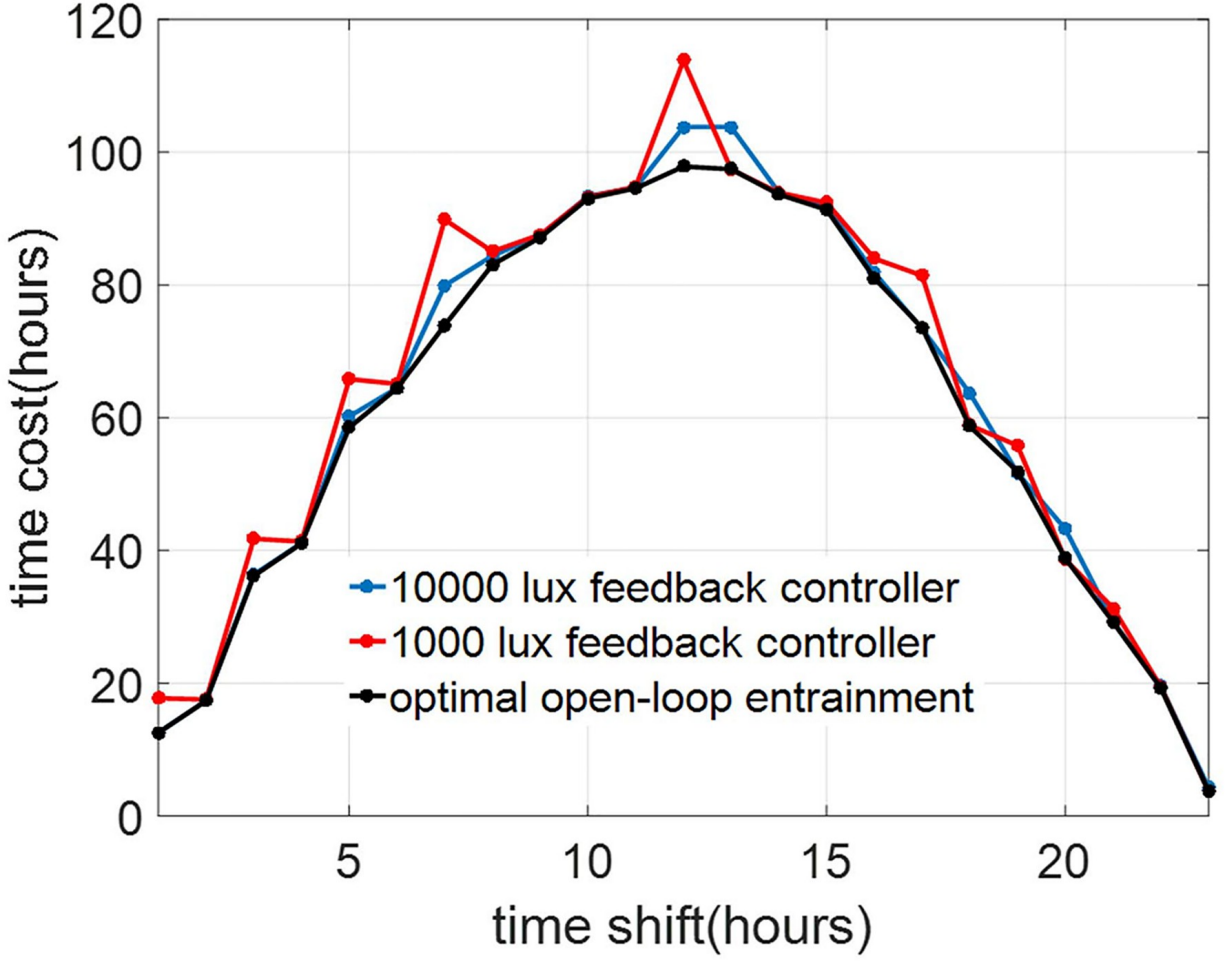
Performance of the optimal feedback controllers trained using data from the cases of circadian light intensities of 1000 and 10000 lux (red and blue curves, respectively) applied to the case of circadian light intensity of 5000 lux. Observe that the feedback controllers perform quite well compared to the actual optimal solutions (black curve).

We train several feedback controllers for different *I*_max_ values. As shown in Fig 14, there are significant differences between these controllers. To evaluate their generalizability, we use the learned feedback controllers with light inputs with different *I*_max_. Specifically, we evaluate the performance of the feedback controllers that are learned using data with *I*_max_ = 10000 lux and *I*_max_ = 1000 lux on circadian light source with *I*_max_ = 5000 lux. The results are shown in Fig 17, which shows that these controllers can still perform well under the change in *I*_max_. For example, the feedback controller for *I*_max_ = 10000 lux results in entrainment times that are less than 10 hours longer compared to the optimal open-loop solutions.

## 7 Discussion and conclusions

In summary, we developed a method for constructing an optimal feedback controller for the time-optimal entrainment problem for the Jewett-Forger-Kronauer (JFK) circadian rhythm model. Our approach is based on the calculus of variations, which enables us to formulate local optimality conditions for the solutions. It also enables us to define a gradient descent algorithm based on the local optimality criteria.

### Comparison of our method and the method in [22]

The optimal entrainment results from our method and the method in [22] demonstrate that our method works as well as the method in [22] in minimizing the entrainment time. The optimal solutions found in this paper demonstrate that we do not encounter singular arcs. In other words, the optimal light input for the minimum-time entrainment of the JFK model is bang-off. The method in [22] is less complex because it has fewer decision variables (i.e., the switching times), but the assumption that the minimum-time optimal light input is bang-off does not always hold, as shown in [19] and [30], due to the existence of the singular arc in the optimal solution. Our method is more general because it does not assume bang-off solutions and, then, could find an optimal solution that contains some singular arcs.

### On the relevance of lower-order circadian rhythm models

Because of the local optimality property, finding the global optimum would require us to search the solution space exhaustively for solutions that satisfy these conditions. While this procedure is not feasible for the full order JFK model, we demonstrate that it can be performed successfully on reduced-order models that we discuss in this paper. Further, the resulting optimal solutions can be used as initial guesses for the optimal solution of the full order model and then improved using the gradient descent algorithm. Therefore, effectively we use the lower-order models as surrogates to the full order model to warm start the gradient descent search algorithm for the optimal solutions.

### Impacts of model reduction on the TOE solutions

Two simplified versions of the JFK model have been discussed in this paper: (i) the 2nd-order model that is the result of ignoring the dynamics of the Process-L, (ii) the 1st-order model resulting from ignoring the dynamics of both the Process-L and the amplitude of the circadian oscillation. We evaluate the impacts of the model reduction on the solutions of the TOE problems by replaying the solutions for the lower-order models on the higher-order ones. The results indicate that for low light intensity, the solutions from the second-order model are practically the same as those from the full order model. Meanwhile, the solutions from the first-order model differ more significantly from those from the second-order model. This suggests that the amplitude dynamics is relatively more important than the Process-L dynamics in solving the TOE problems. Another indicator of the importance of the amplitude dynamics is the fact that in many optimal solutions for the second and full order models the oscillation amplitude is quenched along the way to reach entrainment. This is shown, e.g., in Fig 6 (bottom) and Fig 7 (bottom).

### Potential impacts of amplitude suppression in circadian oscillator

The amplitude suppression phenomenon had been discussed in [29], whose experimental data showed that both the amplitude and phase of the circadian temperature were dramatically changed by the bright light pulses (7000∼12000 lux). This helps explain why the circadian oscillator is dramatically quenched in *I*_max_ = 10000 lux but almost remains on the limit cycle when *I*_max_ = 100 lux during the minimum-time entrainment processes. The amplitude suppression in the circadian oscillator is closely linked to the minimum-time entrainment with bright light in this paper and previous literature. However, CBT amplitude suppression may also have potential connections with health-related issues: the experimental results in [31] indicates that the CBT rhythm tends to flatten with increasing age. The work in [32] also demonstrates that the quenched amplitude of the CBT is closely connected with sleep disruption. Therefore, the entrainment under very bright light with potential sleep disorders may be not suitable for every individual.

### Robustness of the optimal feedback controller

We use a machine learning algorithm to learn an optimal feedback controller for the (full order) TOE problem using data collected from the optimal open-loop trajectories, which are computed using the approach above. In general, we discover that the optimal feedback controller depends on *I*_max_, the maximum circadian light intensity that is used for entrainment. We subsequently train separate optimal feedback controllers for three different values of *I*_max_. However, we also demonstrate that the trained optimal controller is robust to some variations in *I*_max_. Further, the trained feedback controllers depend only on the states of the circadian oscillator, but not on the state of the Process-L. We find that the state of the Process-L has little effect on controller performance.

The minimum-time optimal light strategies proposed in this paper can be implemented on the entraining travelers if the initial circadian states and local time in the destination are known. Although the light intensities of most outdoor lights are highly noisy and maintaining the same lux for hours could be unrealistic, some lighting devices (re-timer and light therapy glasses) and indoor light (the light in the hospital rooms and offices) can be used for offering stable light inputs. The robustness of entrainment against the variation in the light intensity and circadian states is improved by using the feedback entrainment strategy in Section 6, which requires the real-time measurement of the circadian states, i.e., core body temperature. For this purpose, some portable sensors had been developed for instantaneous measurements of the core body temperature [33]. The feedback implementation of the entrainment process is enabled by these sensors.
